# Bioassay-Guided Evaluation and LC-MS/MS Characterization of Five *Limonia acidissima* L. Plant Parts: A Multitarget Approach Against Acne Vulgaris

**DOI:** 10.3390/molecules31183234

**Published:** 2026-09-13

**Authors:** Ika Maruya Kusuma, Berna Elya, Herman Suryadi, Iskandarsyah Iskandarsyah, Mariska Eka Putri, Fadilah Fadilah, Ummu Mastna Zuhri, Najihah Mohd Hashim

**Affiliations:** 1 Faculty of Pharmacy, Universitas Indonesia, Depok 16424, West Java, Indonesia; ika.maruya51@ui.ac.id (I.M.K.); zuhri.u.m@gmail.com (U.M.Z.); 2 Department of Medical Chemistry, Faculty of Medicine, Universitas Indonesia, Jakarta 10430, Indonesia; fadilah.msi@ui.ac.id; 3 Department of Pharmaceutical Chemistry, Faculty of Pharmacy, Universiti Malaya, Kuala Lumpur 50603, Malaysia

**Keywords:** *Limonia acidissima*, antibacterial, antioxidant, 15-lipoxygenase, acne vulgaris

## Abstract

Acne vulgaris is a multifactorial inflammatory disorder involving bacterial colonization, oxidative stress, and lipid-mediated inflammation, necessitating a comprehensive multitarget approach. This study evaluated the anti-acne potential of five morphological parts of *Limonia acidissima* L. using an integrated in vitro and in silico approach. Ultrasound-assisted extracts were evaluated for antibacterial activity against *Cutibacterium acnes* and *Staphylococcus epidermidis*, antioxidant activity using DPPH and ABTS assays, and total phenolic and flavonoid contents (TPC and TFC). TOPSIS was applied to minimize selection bias and identified the leaf extract as the optimal candidate (closeness coefficient = 0.88325). LC-MS/MS tentatively identified major metabolites, including vitexin, quercetin, cyanidin-3-O-galactoside, caffeate, methyleugenol, indole, and linolenic acid. Molecular docking predicted favorable interactions of linolenic acid with *C. acnes* KAS III (Rerank Score = −78.470) and 15-lipoxygenase (15-LOX) (Rerank Score = −101.025), as well as cyanidin-3-O-galactoside with *S. epidermidis* (Rerank Score = −109.588) and KEAP1 (Rerank Score = −132.253). SwissADME analysis further suggested favorable drug-likeness and skin-permeation profiles. Collectively, these findings support the potential of *L. acidissima* leaf extract as a promising multitarget anti-acne candidate.

## 1. Introduction

Acne vulgaris is a chronic skin illness that is highly prevalent and has a broad impact on public health. Globally, more than 85% of adolescents and young adults experience acne vulgaris with varying degrees of severity. In Indonesia, this disease is among the three most common dermatological cases, with a 46% increase in cases at the Cosmetic Dermatology Clinic at Dr. Cipto Mangunkusumo National General Hospital (RSCM) [1]. The high incidence of acne vulgaris in tropical regions such as Indonesia is associated with hot and humid environmental conditions that promote the growth of *Cutibacterium acnes* and *Staphylococcus epidermidis* [2]. Beyond skin lesions, acne vulgaris may also result in psychological disturbances, including anxiety, depression, and reduced quality of life, highlighting the need for effective and safe treatment strategies [3].

Current acne management strategies primarily rely on antibacterial and anti-inflammatory therapies that act directly on acne lesions [4]. However, the widespread and prolonged use of topical antibiotics such as clindamycin may contribute to the development of bacterial resistance in *C. acnes*. It can cause local adverse effects, including erythema, desquamation, skin dryness, and burning sensations [5]. Multitarget therapeutic approaches combining antibacterial, antioxidant, and anti-inflammatory activities have gained increasing attention in acne treatment. This trend reflects the multifactorial pathogenesis of acne vulgaris, which involves not only bacterial colonization but also oxidative stress and complex inflammatory responses. Multitarget therapies are considered more effective than single-target approaches. In recent years, studies on medicinal plants have demonstrated that phytochemicals can simultaneously exert antibacterial, antioxidant, and anti-inflammatory effects, offering a more comprehensive therapeutic strategy for acne management.

The potential of plant-based multitarget anti-acne therapies has been highlighted in both experimental studies and systematic reviews. Phrompanya et al. [6] reported that different parts of *Miliusa velutina* possess distinct antioxidant, antibacterial, and anti-inflammatory activities. The stem bark extract exhibited the highest antioxidant capacity, which correlated with elevated phenolic and flavonoid contents, whereas the leaf extract demonstrated the strongest anti-inflammatory activity. All extracts showed inhibitory effects against acne-causing bacteria. Meanwhile, a systematic review by Kaweesa et al. [7] on *Psorospermum febrifugum* revealed that the antibacterial, anti-inflammatory, and antioxidant activities of medicinal plants are strongly influenced by the chemical structures of their bioactive compounds, which determine their affinity toward molecular targets. Together, these studies show that the main mechanisms behind plant-based anti-acne treatments are antioxidant, antibacterial, and anti-inflammatory activities. The distribution of secondary metabolites among plant sections, the content of phenolic and flavonoid compounds, and the structure-activity relationships of bioactive compounds all affect the effectiveness of these treatments.

Both publications indicate that antioxidant, antibacterial, and anti-inflammatory activities are key mechanisms in the development of plant-based anti-acne therapies. These biological activities are correlated with phenolic and flavonoid content, which contribute to preventing the growth of bacteria, modulating oxidative stress, and suppressing skin inflammation. Furthermore, the plant part used also influenced therapeutic activity because of differences in the distribution of secondary metabolites. However, each approach has its own limitations. Experimental studies provide direct evidence of biological activity but are often limited in identifying specific molecular targets and underlying mechanisms. In contrast, previous reviews summarize the available evidence but cannot directly validate the biological relevance of the proposed mechanisms. Although Phrompanya et al. [6] identified a correlation between phenolics and biological activity, and Kaweesa et al. [7] demonstrated the structure of active compounds and their affinities, to date, no study has integrated molecular docking predictions of 15-LOX (15-lipoxygenase) inhibitors with in vitro biological activity in *C. acnes* and *S. epidermidis* in *L. acidissima*. This study was conducted to fill this gap by integrating LC-MS/MS and molecular docking results with a specific target 15-LOX inhibitor, as well as its antibacterial and antioxidant activities in vitro.

The 15-LOX enzyme was chosen because it contributes to sebum lipid oxidation, catalyzing the oxidation of fatty acids, resulting in the production of lipid mediators that not only contribute to the initiation and progression of inflammation in acne but also play a role in the inflammatory mechanisms necessary for tissue protection and repair [8]. Therefore, integrating biological assays with computational screening through molecular docking is expected to provide a thorough comprehension of the therapeutic potential of plant-derived anti-acne agents.

*L. acidissima* (family Rutaceae) was selected based on previous monotarget studies demonstrating its antioxidant potential. The leaf extract exhibited an IC_50_ value of 10.445 μg/mL, whereas the stem bark extract showed an IC_50_ value of 17.4 μg/mL in the DPPH assay [9,10]. In addition, the ethanol extract of the leaves was reported to contain a total phenolic content of 63.84%. The presence of an inhibition zone of 14–17 mm also demonstrates the activity of *L. acidissima* fruit peel against *S. aureus* [11]. In the *L. acidissima* fruit pulp, anti-inflammatory activity was also demonstrated through an albumin denaturation test with an inhibition percentage reaching 74.55% [12]. However, there has been no integrated evaluation of this plant. This study provides in vitro validation of the antibacterial and antioxidant assays, as well as the integrated in silico analysis of *L. acidissima* against the antibacterial, antioxidant, and 15-LOX target, which has not yet been evaluated in *L. acidissima*. Furthermore, compounds in the best extracts were identified by LC-MS/MS, with direct correlation through molecular docking, a finding rarely reported in Rutaceae, particularly *L. acidissima*.

Unlike previous studies, which generally focused on a single biological activity or a single plant part, this study compared various plant parts: leaves, fruit pulp, fruit peel, twigs and bark. This selection was designed to map the relationship between the chemical constituents and biological activity of *L. acidissima* to identify the most potent in vitro antibacterial and antioxidant compounds. In addition to in vitro biological testing, this study integrated an in silico approach to target inflammatory lipoxygenase.

## 2. Results and Discussion

### 2.1. Extraction Yield and Phytochemical Screening

The extraction yield of the investigated plant parts was in the order of fruit pulp (highest), stem bark, leaves, fruit peel, and twigs (Table 1). The UAE method enhances extraction efficiency, with extraction using ethanol at 50 °C for 40 min, resulting in higher yields and improved biological activity. This observation is consistent with previous findings showing that leaf extraction using UAE yielded 13.50%, which was higher than that obtained using maceration with 96% ethanol (11.83%) [11]. Similarly, Yusnaini et al. [13] reported a yield of 5.52% for fruit pulp extracted by maceration for 3 × 24 h. In contrast, the UAE method employed in this study produced a substantially higher yield of 25.58% within only 40 min at 50 °C. The higher extraction yields obtained using UAE may be attributed to the cavitation effect generated by ultrasonic waves, which enhances solvent penetration into plant tissues and facilitates the release of intracellular compounds. Overall, the results show that using ethanol as the extraction solvent at 50 °C for 40 min is an efficient approach for extracting bioactive constituents from *L. acidissima*. Phytochemical screening results indicate that extracts from all parts of the *L. acidissima* plant contain alkaloids, flavonoids, tannins, and saponins. These findings align with previous studies reporting the presence of these compounds in the fruit, bark, and leaves of *L. acidissima* [14,15].

The relationship between phytochemical composition and the biological activity of the alkaloids, flavonoids, tannins, and saponins in *L. acidissima* contributes to its antibacterial, antioxidant, and anti-inflammatory activities. Alkaloids inhibit cell wall synthesis, increase membrane permeability, and disrupt bacterial nucleic acid and protein synthesis [16]. Flavonoids and tannins are antioxidants that donate electrons or hydrogen atoms to neutralize free radicals, and saponins help protect cells from oxidative damage [15]. Furthermore, flavonoids, alkaloids, and saponins inhibit the production of pro-inflammatory cytokines and inducible nitric oxide synthase (iNOS) and thus show anti-inflammatory effects. Flavonoids modulate Nuclear Factor Kappa B (NF-κB) and Mitogen-Activated Protein Kinase (MAPK) signaling pathways, whereas alkaloids and saponins help suppress oxidative stress, which contributes to the inflammatory response [17,18]. Therefore, the variation in biological activity among different parts of the *L. acidissima* plant is likely linked to the synergistic effects of alkaloids, flavonoids, tannins, and saponins, as well as the composition and concentration of bioactive compounds in the extracts.

### 2.2. Total Phenolic Content (TPC) and Total Flavonoid Content (TFC)

The highest TPC was observed in the leaf extract (66.11 ± 0.68 mg GAE/g extract), which was not significantly different from that of the fruit peel extract (61.58 ± 1.75 mg GAE/g extract). Similarly, the highest TFC was recorded in the leaf extract (197.36 ± 3.06 mg QE/g extract), followed by the fruit pulp extract (25.33 ± 0.14 mg QE/g extract) (Table 2). These findings indicate that the leaf extract contains the highest levels of phenolic and flavonoid compounds among the plant parts investigated. Compared with previous research, namely, the maceration method, leaf extract from Bima in West Nusa Tenggara, Indonesia, had a TPC value (14.63 mg GAE/g) and TFC (113.9 mg QE/g), whereas the UAE method had a higher value [9,10]. Phenolic and flavonoid compounds exhibit antioxidant activity through their ability to scavenge free radicals. This activity is attributed to the number of hydroxyl groups and the aromatic structure of phenolic compounds. In contrast, flavonoids, a class of phenolic compounds, exert antioxidant activity through mechanisms involving hydrogen atom donation and transition metal ion chelation [19,20].

### 2.3. Antibacterial Activity of Limonia acidissima Plant Parts

The antibacterial activity of *L. acidissima* extract against *C. acnes* and *S. epidermidis* was influenced by the concentrated extracts from all plant parts tested. All extracts inhibited the growth of both bacteria at concentrations of 3.125%, 6.25%, 12.5%, 25%, 50%, and 75%, although the extent of inhibition varied among plant parts (Table 3 and Table 4, Figure S1). Increasing the extract concentration consistently increased the diameter of the inhibition zone. This indicates that higher concentrations probably have more active compounds that can suppress the growth of bacteria. Although all extracts showed measurable antibacterial activity, their inhibition zones remained smaller than those produced by the positive control clindamycin. This difference is considered because plant extracts contain a complex mixture of compounds with varying mechanisms of action, whereas clindamycin is a pure antibiotic with a specific mechanism of action [21]. Nevertheless, the antibacterial activity observed in all extracts indicates active chemical compounds with potential anti-acne properties.

The pathogenesis of acne is closely related to the antibacterial effects on *C. acnes* and *S. epidermidis*. *C. acnes* participates in acne pathogenesis by producing lipase, which hydrolyzes sebum triglycerides into free fatty acids that induce follicular hyperkeratinization and inflammation [22,23]. Furthermore, *C. acnes* can activate the Toll-like receptor 2 (TLR2)-mediated signaling pathway, resulting in the liberation of pro-inflammatory cytokines, such as interleukin (IL-1β, IL-6, IL-8) and tumor necrosis factor-alpha (TNF-α). *S. epidermidis* is a commensal member of the normal skin microbiota, but dysbiosis can cause some strains to become opportunistic pathogens that can produce biofilms and enhance the inflammatory response [23]. Therefore, inhibition of these two bacteria might be beneficial for the reduction in bacterial colonization and inflammation in acne lesions.

The phenolic and flavonoid contents of the extract have been widely reported to exert antibacterial effects through various mechanisms. These compounds can disrupt bacterial cell membrane integrity, increase membrane permeability, denature membrane proteins, inhibit essential metabolic enzymes, disrupt nucleic acid synthesis, and induce leakage of intracellular components, ultimately leading to bacterial cell death [23,24]. Furthermore, phenolic and flavonoid compounds have been reported to inhibit the production of Extracellular polymeric substances (EPS), thereby reducing bacterial adhesion and biofilm formation [23]. Although biofilm inhibition was not directly evaluated in this study, the observed antibacterial activity suggests that *L. acidissima* extract may have antibiofilm potential that warrants further investigation. Based on the diameter of the inhibition zone, the antibacterial activity of the extract against *C. acnes* and *S. epidermidis* can be classified as moderate, with the inhibition zone generally ranging from 5 to 10 mm [24], even at the lowest concentration tested. This finding is consistent with a previous report where the ethyl acetate extract of *L. acidissima* fruit peel at a concentration of 6.25% produced an average inhibition zone of 9.95 mm against *C. acnes*, which was also categorized as moderate [25]. Comparable antibacterial activity supports the potential of *L. acidissima* as a natural anti-acne agent.

### 2.4. Antioxidant Activity of Limonia acidissima Plant Parts

The 2,2-Diphenyl-1-picrylhydrazyl (DPPH) and 2,2′-Azino-bis(3-ethylbenzothiazoline-6-sulfonic acid) (ABTS) tests are used to determine the antioxidant capacity of an extract based on its ability to donate hydrogen atoms or electrons to neutralize free radicals. The DPPH method measures the extract’s ability to scavenge the stable radical DPPH, whereas the ABTS method measures the ability to scavenge the radical ABTS cation. Antioxidant activity is expressed as the IC_50_ value. The lower the IC_50_ value, the stronger the antioxidant capacity because the concentration required to inhibit 50% of free radicals is lower [26].

Based on the antioxidant test results presented in Table 5, extracts from various parts of *L. acidissima* exhibit varying levels of antioxidant activity as determined by the DPPH and ABTS methods. The DPPH results showed that the twig extract showed the highest antioxidant activity, with an IC_50_ value of 50.22 ± 2.99 µg/mL, followed by the stem bark extract (71.64 ± 10.00 µg/mL) and leaf extract (78.16 ± 13.37 µg/mL). In contrast, the fruit peel extract (219.70 ± 36.92 µg/mL) and fruit pulp (228.52 ± 35.43 µg/mL) showed relatively weaker antioxidant activity. The ABTS test results showed that the twigs exhibited the highest antioxidant activity, with an IC_50_ of 73.290 ± 0.76 µg/mL, followed by the bark (76.90 ± 0.69 µg/mL) and leaves (78.42 ± 0.43 µg/mL). Conversely, the fruit peel had lower activity (88.50 ± 1.21 µg/mL), whereas the fruit pulp had the weakest antioxidant activity, with an IC_50_ value of 799.21 ± 17.16 µg/mL.

The results showed that the antioxidant activity of *L. acidissima* was influenced by the plant part. In the DPPH method, the twig extract had the highest antioxidant capacity, followed by the stem bark and leaves, whereas the fruit peel and fruit pulp extracts showed much lower activity. In the ABTS test, twig, stem bark, and leaf extracts also gave relatively low IC_50_ values, whereas fruit pulp extract had the lowest activity. Significant differences between plant parts (*p* < 0.05) indicate that the distribution of bioactive metabolites that act as antioxidants is not uniform across *L. acidissima* plant parts.

All extracts demonstrated the ability to scavenge free radicals, although their activity was lower than that of the positive control, ascorbic acid, which showed IC_50_ values of 8.32 ± 0.08 µg/mL for DPPH and 5.72 ± 0.05 µg/mL for ABTS. The very high activity of ascorbic acid is due to its pure antioxidant activity through efficient electron transfer and hydrogen atom donation mechanisms [27].

Oxidative stress is one mechanism in the pathogenesis of acne. Colonization with *C. acnes* increases the formation of reactive oxygen species (ROS), which induce lipid peroxidation in sebum, damage cell membranes, and activate inflammatory pathways through transcription factors such as NF-κB. Activation of these pathways increases the production of pro-inflammatory cytokines, including IL-1β, IL-6, IL-8, and TNF-α, thereby exacerbating follicular inflammation [22]. Therefore, the antioxidant activity of *L. acidissima* extract has potential as a supportive anti-acne therapy by reducing ROS levels and inhibiting oxidative stress. This mechanism may help reduce tissue damage and the inflammatory response triggered by *C. acnes*.

The results of TPC and TFC content, when associated with antioxidant activity, do not show a completely linear relationship. The leaf extract had the highest phenolic (66.11 mg GAE/g) and flavonoid (197.36 mg QE/g) content, but the best antioxidant activity was found in the twig extract, although it was still in the same category. Conversely, the fruit peel had a relatively high phenolic content (61.58 mg GAE/g) but exhibited low antioxidant activity. These findings indicate that antioxidant activity is influenced not only by the total amount of phenolics or flavonoids but also by the composition, chemical structure, and type of bioactive compounds contained in the extract.

This is consistent with previous reports that leaf extracts have superior antioxidant activity (IC_50_ = 10.445 μg/mL) compared to fruit peel and fruit pulp extracts, which showed IC_50_ values of 233.16 and 698.44 ppm, respectively [9,28]. Overall, the results suggest that twigs, bark, and leaves are superior sources of antioxidants compared to fruit, thus warranting further research. The relatively high standard deviations of the test results suggest heterogeneity in the phytochemical composition and bioactive characteristics of the plant extracts.

### 2.5. Selection of the Optimal Extract Using TOPSIS

Extracts obtained from different parts of *L. acidissima* were evaluated and ranked using the TOPSIS method based on their phytochemical composition and biological activities. The data were subsequently normalized and weighted to construct the decision matrix. Positive ideal (A^+^) and negative ideal (A^−^) solutions were then determined according to the minimization and maximization criteria (Table 6). The separation distances from the positive ideal solution (D^+^) and negative ideal solution (D^−^) were calculated, followed by the determination of the closeness coefficient (CC*_i_*) values. Based on the CC*_i_* values, the extracts were ranked in the following order: leaf extract (0.88325) > twig extract (0.52321) > stem bark extract (0.52314) > fruit peel extract (0.48878) > fruit pulp extract (0.04387). The larger the CC*_i_* value, the closer the alternative is to the positive ideal solution, and the smaller the CC*_i_* value, the further the alternative is from the positive ideal solution [29].

The TOPSIS results indicated that the leaf extract was the most optimal plant part, with the highest closeness coefficient (CC*_i_* = 0.88325). The ranking may be attributed to its high total flavonoid content, high total phenol content, and strong antioxidant activity. Furthermore, the leaf extract exhibited considerable antibacterial activity against ACE-associated bacteria [30]. These findings suggest that the leaf extract possesses the most favorable combination of phytochemical constituents and biological activities among all tested parts of *L. acidissima*, as reflected by its highest CC*_i_* value, indicating the closest proximity to the ideal solution (Table 7) [31].

### 2.6. LC-MS/MS Characterization of Bioactive Compounds

**Figure 1:**
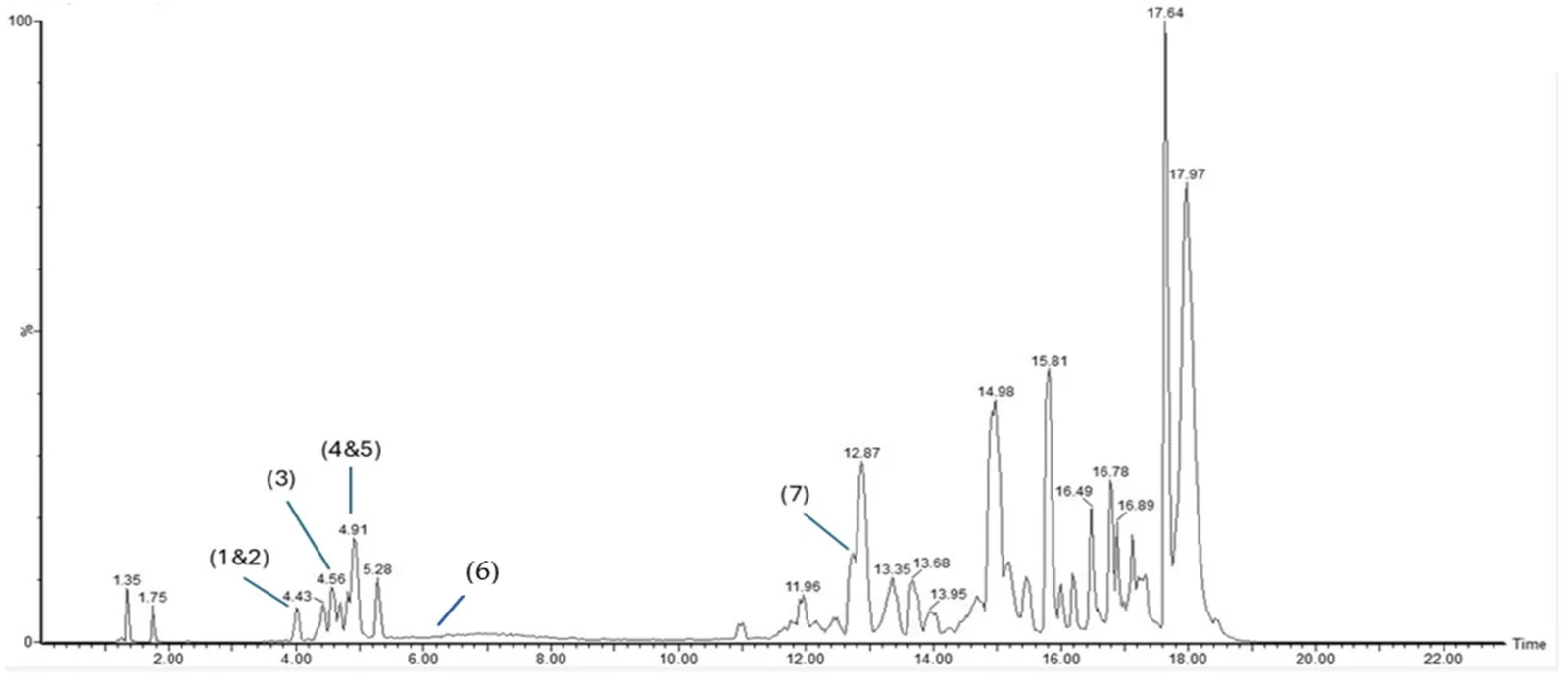
Total ion chromatogram of LC-MS/MS leaf extract *L. acidissima* in positive ionization mode with peak numbering of key metabolites.

Based on the TOPSIS evaluation, the ethanol extract of *L. acidissima* leaves demonstrated the most optimal bioactivity profile, directing the extract as the subject for high-resolution LC-MS/MS analysis. The dereplication process putatively identified 159 constituents (Supplementary Table S1), with seven constituents previously measured in *L. acidissima* (Table 8, Figure 1), with annotation confidence at MSI Level 3 [32]. The major constituents showed diverse chemical classes, including flavonoids, phenolic acids, alkaloids, and lipids. Chromatographic profiling revealed that the predominant peak, eluting at a retention time of 4.92 min, corresponded to vitexin.

Previously reported seven compounds detected in *L. acidissima* leaf extract exhibit antioxidants, antibacterial, and anti-inflammatory activities related to acne pathogenesis. The ability of caffeate, quercetin, vitexin, and cyanidin-3-O-galactoside to inhibit oxidative stress includes scavenging reactive oxygen species (ROS), suppressing NF-κB pathway activation, and reducing pro-inflammatory cytokine levels [33,34,35]. Indole contributes to the antioxidant potential of the extract through its reported free radical scavenging and oxidative stress regulating properties, while methyleugenol contributes to its anti-inflammatory activity by suppressing inflammatory mediators involved in the response to *C. acnes* [36,37]. Moreover, the diversity of these annotated compounds reveals the extract’s putative multitarget efficacy. Flavonoids such as quercetin and vitexin are extensively documented for their multiple effects, acting both as potent intracellular ROS scavengers and potent inhibitors of pro-inflammatory cytokine cascades [33,38]. Concurrently, phenolic derivatives like caffeate contribute significantly to antibiofilm activities, an important mechanism for mitigating *C. acnes* adherence and colonization [39]. Furthermore, the presence of a lipid group (linolenic acid) offers an additional therapeutic potential by restoring the epidermal barrier function and modulating sebaceous lipogenesis [40].

### 2.7. Molecular Docking Analysis of Antibacterial, Antioxidant, and 15-Lipoxygenase Targets

LC-MS/MS analysis identified compounds in *L. acidissima* leaves as indole, caffeic acid, cyanidin-3-O-galactoside, vitexin, quercetin, methyleugenol, and linolenic acid. These phytochemicals may be responsible for the antibacterial, antioxidant, and anti-inflammatory activities. To support these findings, molecular docking was performed to evaluate the interaction of the identified compounds with selected targets related to *C. acnes*, *S. epidermidis*, antioxidant activity, and inflammation.

**Figure 2:**
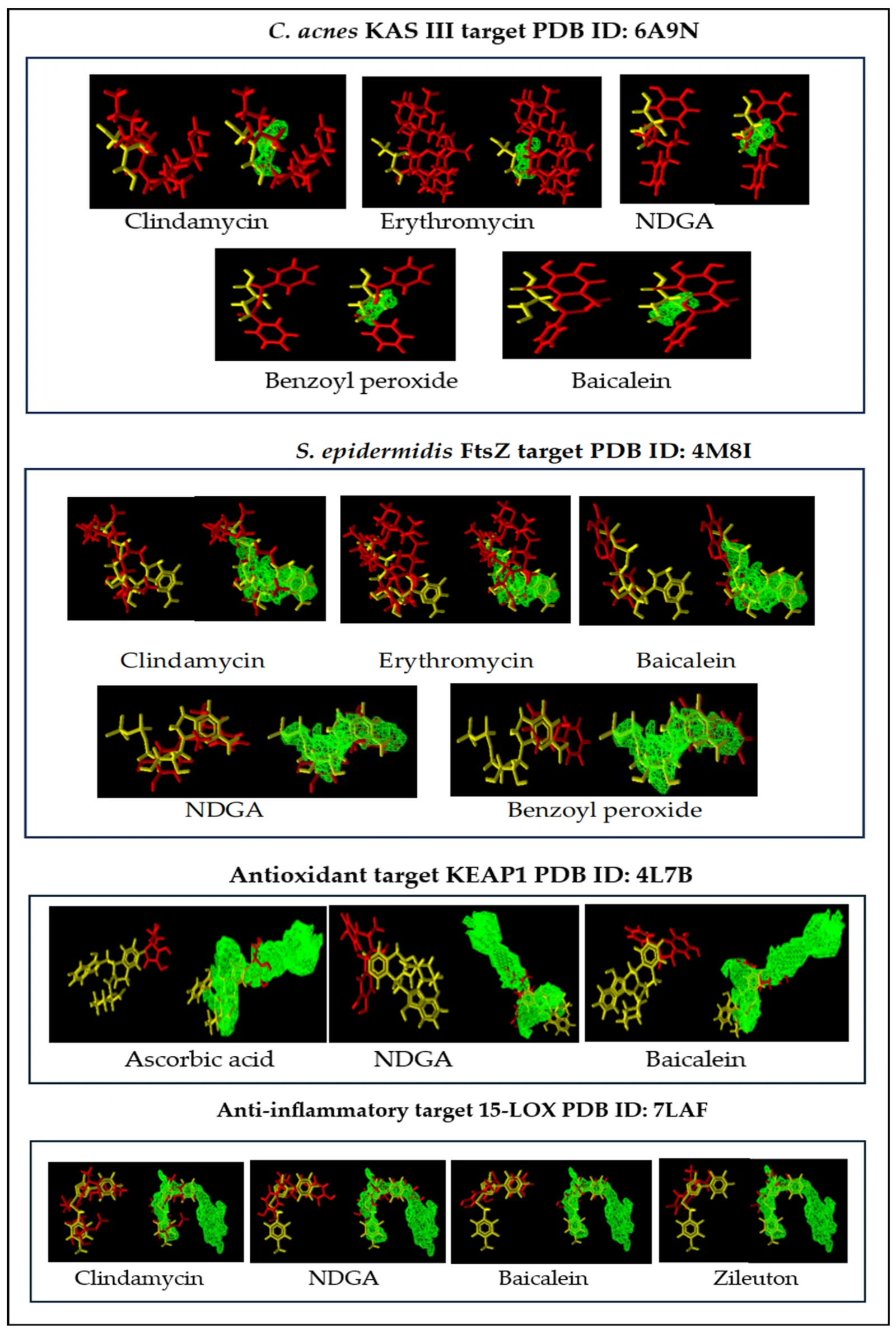
Reference ligands and target protein structures used for molecular docking analysis.

In the initial stage of ligand structure preparation, the best cavity is selected from several cavities identified in the target protein. The position of the cavity is determined by considering the binding site of the reference ligand, as shown in Figure 2. In this study, the reference ligands used were antibacterial (NDGA, baicalein, erythromycin, benzoyl peroxide, and clindamycin), antioxidant (Ascorbic acid, NDGA, baicalein), and anti-inflammatory (NDGA, baicalein, zileuton, and clindamycin). These reference ligands were selected for molecular docking studies. NDGA and baicalein, together with established anti-acne agents, namely erythromycin, benzoyl peroxide, and clindamycin, were used as reference compounds to evaluate the predicted antibacterial activity. Meanwhile, ascorbic acid, NDGA, and baicalein were used as reference compounds to evaluate the predicted antioxidant activity.

**Figure 3:**
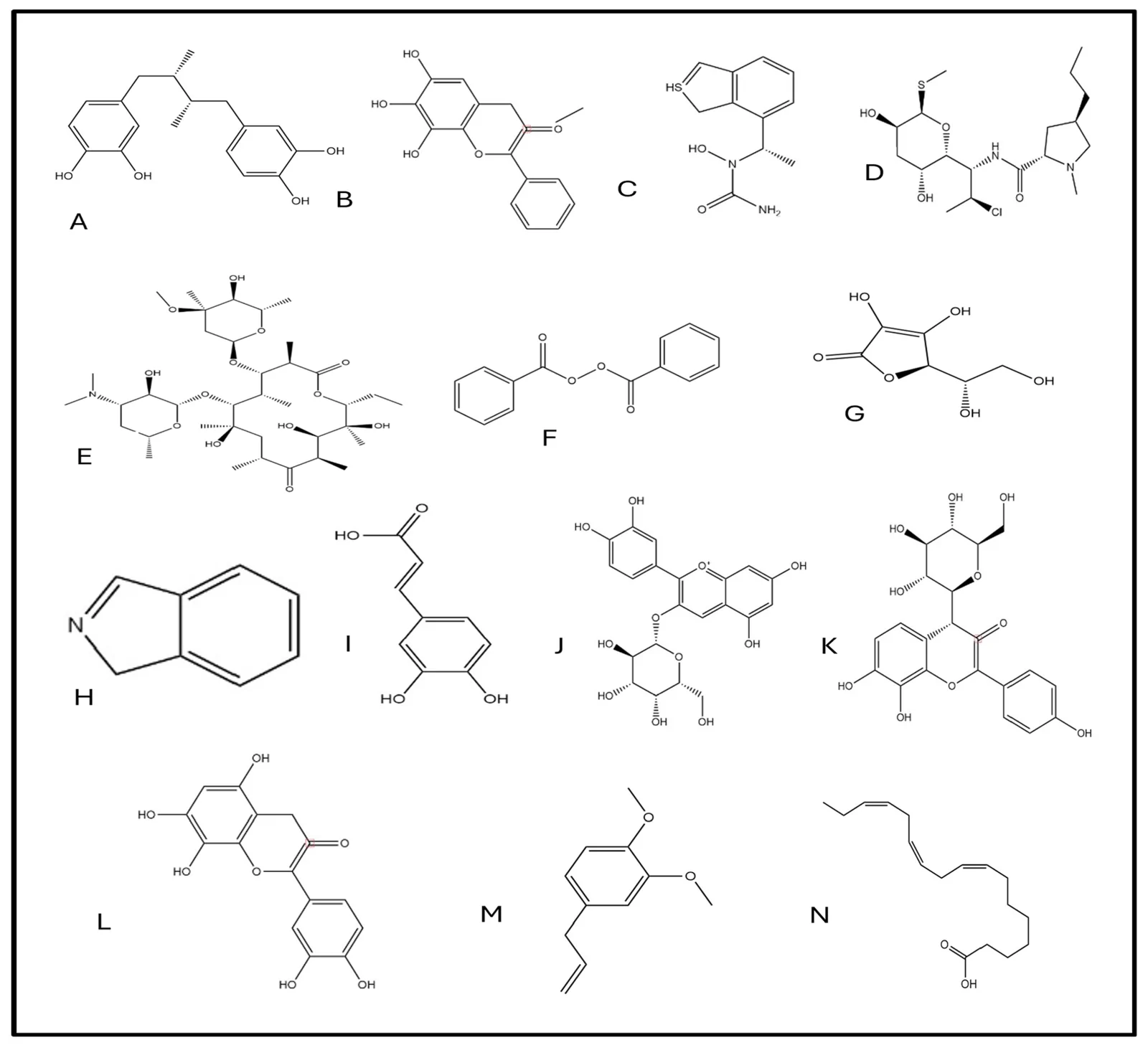
Two-dimensional (2D) chemical structures: (**A**) NDGA, (**B**) Baicalin, (**C**) Zileuton, and (**D**) Clindamycin, (**E**) Erythromycin, (**F**) Benzoyl peroxide, (**G**) Ascorbic acid as reference ligands; (**H**) Indole, (**I**) Caffeate, (**J**) Cyanidin-3-O-galactoside, (**K**) Vitexin, (**L**) Quercetin, (**M**) Methyleugenol, and (**N**) Linolenic acid as test ligands.

NDGA and zileuton were used as reference ligands because they are lipoxygenase inhibitors with reported inhibitory activity against the lipoxygenase enzyme and are widely used to evaluate the binding affinity of lipoxygenase enzyme compounds [41,42]. Bacalein was chosen for its inhibitory activity against lipoxygenase, particularly the 15-LOX pathway, as a natural compound [43]. Meanwhile, clindamycin is an anti-acne drug that is widely used as a comparison for clinical treatment to evaluate the potential of test compounds in inflammation caused by acne [44]. The use of clindamycin as a comparison provides an overview of the binding affinity of test compounds to receptors related to the inflammatory process of acne. The entire docking process, both for reference and test ligands, was performed in the same cavity to ensure consistency of binding sites and interaction patterns. Figure 3 shows the structure of each compound.

The LC-MS/MS-identified compounds showed different predicted binding affinities toward the *C. acnes* KAS III target within the selected binding cavity. Among the identified compounds, linolenic acid exhibited the most favorable predicted interaction with the *C. acnes* KAS III target, as evidenced by a Rerank Score of −78.470, indicating the most favorable predicted binding score among the tested metabolites. This value was close to that of the clinical anti-acne reference drug clindamycin (−79.224), and was more favorable than those of quercetin (−74.807), vitexin (−69.562), caffeate (−63.329), cyanidin-3-O-galactoside (−54.933), methyleugenol (−54.920), and indole (−39.202). Linolenic acid interacted with Asp180, His10, Ser179, and Pro8, whereas clindamycin interacted with Ile136, Asn142, Gly139, His10, Pro8, and Arg137 (Table 9, Figure S2). Notably, linolenic acid and clindamycin shared two interaction residues, His10 and Pro8, suggesting that both ligands occupied or interacted with a partially overlapping region of the KAS III binding cavity. The antibacterial potential of linolenic acid is supported by previous experimental studies demonstrating its antibacterial activity against Gram-positive bacteria [45]. In addition, quercetin has been experimentally reported to exhibit antibacterial activity against *C. acnes*, with a minimum inhibitory concentration (MIC) of 31.25 μg/mL, further supporting its potential antibacterial activity [46].

Against the *S. epidermidis* target, the identified compounds generally showed more favorable predicted interactions than those observed for the *C. acnes* target. The predicted interaction with the *S. epidermidis* target was observed for Cyanidin-3-O-galactoside, which exhibited the most favorable Rerank Score (−109.588), followed by vitexin (−99.830), linolenic acid (−98.356), quercetin (−97.154), caffeate (−77.798), methyleugenol (−70.467), and indole (−51.726) (Table 9). Interestingly, cyanidin-3-O-galactoside showed a more favorable Rerank Score than the reference antibacterial compounds clindamycin (−96.592) and erythromycin (−61.676), suggesting a highly favorable predicted interaction within the selected binding cavity. Cyanidin-3-O-galactoside shares Thr133, Met105, and Gly104 with clindamycin, while it shares Arg143 and Ala103 with NDGA (Figure S3). Notably, Glu139 and Met105 are common interaction residues among cyanidin-3-O-galactoside and the reference compounds clindamycin, NDGA, and erythromycin, suggesting that these residues may contribute to ligand recognition within the selected FtsZ binding cavity. Previous experimental studies have demonstrated the potential antibacterial activity of cyanidin-3-O-glucoside against other acne-causing bacteria [47].

Docking analysis against KEAP1 (PDB ID: 4L7B) further supported the antioxidant potential of the identified compounds. Cyanidin-3-O-galactoside exhibited the most favorable Rerank Scores (−132.253), followed by linolenic acid (−98.553), quercetin (−99.745), vitexin (−93.461), and caffeate (−69.308) (Table 9). Notably, all of these compounds showed more favorable predicted ligand–protein interactions than the reference compound ascorbic acid (−66.587), as indicated by their more negative Rerank Scores. Cyanidin-3-O-galactoside interacts with residues including Asn414, Ser363, Gly364, Val604, Leu365, Gly603, Leu557, Gly509, Ser508, and Arg415 (Figure S4). Gly364 was a common interacting residue shared by cyanidin-3-O-galactoside, quercetin, linolenic acid, vitexin, and the ascorbic acid reference ligand. In addition, Leu557 was commonly involved in the interactions of cyanidin-3-O-galactoside, quercetin, caffeate, and ascorbic acid. These shared residues may contribute to ligand recognition and binding within the selected KEAP1 binding site. The antioxidant potential of cyanidin-3-O-galactoside is supported by previous experimental evidence, which reported an EC_50_ value of 0.221 mg/mL in the DPPH radical-scavenging assay [48].

Inflammation is a key mechanism in acne pathogenesis. Evaluation of 15-LOX inhibitory activity is widely used to assess the potential of natural anti-inflammatory agents. The 15-LOX enzyme plays a role in the metabolism of arachidonic acid into pro-inflammatory lipid mediators [40]. Inhibiting its activity can reduce the inflammatory response that contributes to acne lesion development. The identified compounds were then compared with reference ligands (NDGA, baicalin, zileuton, and clindamycin), against the 15-LOX enzyme. Based on the Rerank Score, linolenic acid has the highest binding affinity (−101.025), even lower than that of the comparison ligands, namely NDGA (−98.890), baicalin (−79.835), zileuton (−76.540), and clindamycin (−42.009). The more negative the Rerank Score, the more stable the ligand-protein complex formed. While linolenic acid does not form hydrogen bonds, it exhibits excellent affinity through hydrophobic interactions with the amino acid residue Val426. The high affinity of linolenic acid is thought to be related to its structural suitability as an unsaturated fatty acid that can interact with the substrate binding site on the LOX enzyme. Therefore, this compound can be a ligand that binds strongly to 15-LOX. Caffeate and quercetin compounds showed quite good binding affinity to the 15-LOX enzyme with Rerank Scores of −69.890 and −69.707. Both compounds formed hydrogen bonds with key residues, namely, His378, Glu369, Ala416, Ile676, and Leu420, which have similarities with the residues of the reference ligand.

Methyleugenol and vitexin compounds showed moderate affinity with Rerank Scores of −67.932 and −62.943, while indole had low affinity, respectively, −48.478. Although cyanidin-3-O-galactoside formed the strongest hydrogen bond (H-bond energy = −11.345), the positive Rerank Score (6.865) indicates that the formed complex is less stable due to less than optimal binding orientation. Compared to the reference ligand clindamycin, which has a rank score of −42.009 and interacts with residues Leu420, Glu369, His373, and Ile676, caffeate, quercetin, and vitexin compounds have higher binding affinity values and similar interaction residues in the active pocket of the enzyme. Therefore, these three compounds are potential 15-LOX inhibitors. However, based on the binding affinity values of all test compounds, linolenic acid is the most prospective candidate.

15-LOX catalyzes the oxidation of arachidonic acid to lipid mediators that contribute to the inflammatory response; therefore, its inhibition may help reduce inflammation associated with acne lesions. Although linolenic acid exhibited the highest predicted binding affinity toward 15-LOX based on the Rerank Score, its interaction was dominated by hydrophobic bonds, with no hydrogen bonds detected. Linolenic acid showed a distinct interaction pattern within the 15-LOX binding site, including an interaction with Val426 that was not observed for the reference ligands or other compounds. This interaction may be related to the long hydrophobic chain of linolenic acid, which may promote favorable bonds with hydrophobic residues within the lipoxygenase binding pocket. Previous studies have identified Val426 as part of the ligand-binding environment of 15-LOX and indicated that residues surrounding this position contribute to the accommodation of lipid substrates within the active site [49]. In addition, the C-terminal catalytic domain of LOX forms a curved, U-shaped channel that accommodates fatty acid substrates. The 18-carbon chain length of polyunsaturated fatty acids (PUFAs), such as linoleic acid, is well suited to this hydrophobic channel, allowing the substrate to adopt a favorable conformation and position the target carbon atoms in proximity to the non-heme iron cofactor, thereby facilitating hydrogen abstraction [50]. Meanwhile, caffeate, quercetin, and vitexin show residue interaction patterns that are more similar to the reference inhibitor, indicating a more consistent inhibitory mechanism against 15-LOX (Table 9, Figure S5). In addition, these three compounds are known to have antioxidant and anti-inflammatory activities through suppression of reactive oxygen species (ROS), suppression of NF-κB activation, and suppression of pro-inflammatory cytokine production.

### 2.8. Integrated Anti-Acne Mechanism

The antibacterial activity of *L. acidissima* leaf extract against *C. acnes* and *S. epidermidis* demonstrates its ability to inhibit bacteria implicated in acne pathogenesis. This finding was further supported by the molecular docking results. Linolenic acid showed a favorable predicted interaction with the *C. acnes* KAS III target (Rerank Score, −78.470), comparable to clindamycin (−79.224), whereas cyanidin-3-O-galactoside exhibited the most favorable predicted affinity toward the *S. epidermidis* target (−109.588), exceeding those predicted for clindamycin (−96.592), erythromycin (−61.676), and benzoyl peroxide (−86.380). The shared interacting residues between cyanidin-3-O-galactoside and the reference antibacterial ligands suggest that this compound may interact with functionally relevant regions of the bacterial target. In addition, quercetin and caffeate have been reported to possess antibacterial and antibiofilm activities, which may further contribute to the inhibition of bacterial adhesion and colonization [39].

Antibacterial activity may be further complemented by the strong antioxidant activity of the extract observed in the DPPH and ABTS assays. Oxidative stress associated with *C. acnes* colonization can increase reactive oxygen species (ROS) production, promote sebum lipid peroxidation, and activate inflammatory pathways [51]. Consistent with this biological activity, molecular docking against KEAP1 indicated favorable predicted interactions for cyanidin-3-O-galactoside, linolenic acid, quercetin, vitexin, and caffeate. Among these compounds, cyanidin-3-O-galactoside exhibited the most favorable predicted binding affinity (Rerank Score, −132.253) and shared interacting residues with several antioxidant candidates and the ascorbic acid reference ligand. The high phenolic and flavonoid content of the extract, including caffeate, quercetin, vitexin, and cyanidin-3-O-galactoside, may contribute to its radical-scavenging capacity and potential protection against oxidative stress.

In addition, the anti-inflammatory activity provides a complementary mechanism for controlling the progression of acne lesions. Linolenic acid exhibited the most favorable predicted affinity toward 15-LOX (Rerank Score, −101.025), compared with NDGA (−98.890), baicalin (−79.835), zileuton (−76.540), and clindamycin (−42.009). Its interactions were predominantly hydrophobic and involved Val426, whereas caffeate, quercetin, and vitexin interacted with several residues also recognized by the reference ligands. These compounds have also been reported to suppress NF-κB activation and the production of pro-inflammatory cytokines. Collectively, the antibacterial, antioxidant, and anti-inflammatory activities suggest a potential multitarget mechanism of *L. acidissima* leaf extract, in which complementary effects may simultaneously reduce bacterial colonization, oxidative stress, and inflammatory responses. This integrated activity may provide a broader therapeutic rationale than targeting a single pathway alone.

The SwissADME prediction results show that most of the compounds identified in *L. acidissima* leaf extract meet Lipinski’s Rule of Five criteria, indicating good oral bioavailability potential, except for cyanidin-3-O-galactoside (Table 10). Linolenic acid and vitexin compounds slightly exceed the limit set by Lipinski (Log P ≤ 5), but this is still tolerable. Topical application parameters, Log P, topological polar surface area (TPSA), estimated solubility (ESOL), and skin permeation (Log Kp) are all factors that determine skin penetration. Linolenic acid, caffeate, methyleugenol, and indole exhibit favorable profiles for TPSA, water solubility, skin permeation, and Lipinski rules. These compounds are thought to have characteristics that support both topical and oral delivery. Conversely, quercetin, vitexin, and cyanidin-3-O-galactoside have high TPSA and therefore exhibit lower skin permeation. This agrees with previous studies reporting that highly polar flavonoids generally have lower skin permeability than less polar phenolic compounds. Furthermore, compounds with TPSA values < 90 Å^2^ generally have better skin permeability [52]. Overall, the physicochemical properties and SwissADME predictions indicate that methyleugenol, indole, linolenic acid, and caffeate are the compounds with the most potential physicochemical profiles for development as anti-acne agents. While other compounds can contribute bioactivity through multitarget mechanisms, they face limitations in their pharmacokinetic characteristics. This multitarget approach has advantages over treatments that target only one mechanism and indicates that *L. acidissima* leaf extract has further potential. However, these results are still in vitro and in silico testing, and further research is needed to confirm their mechanism of action and effectiveness.

## 3. Materials and Methods

**Figure 4:**
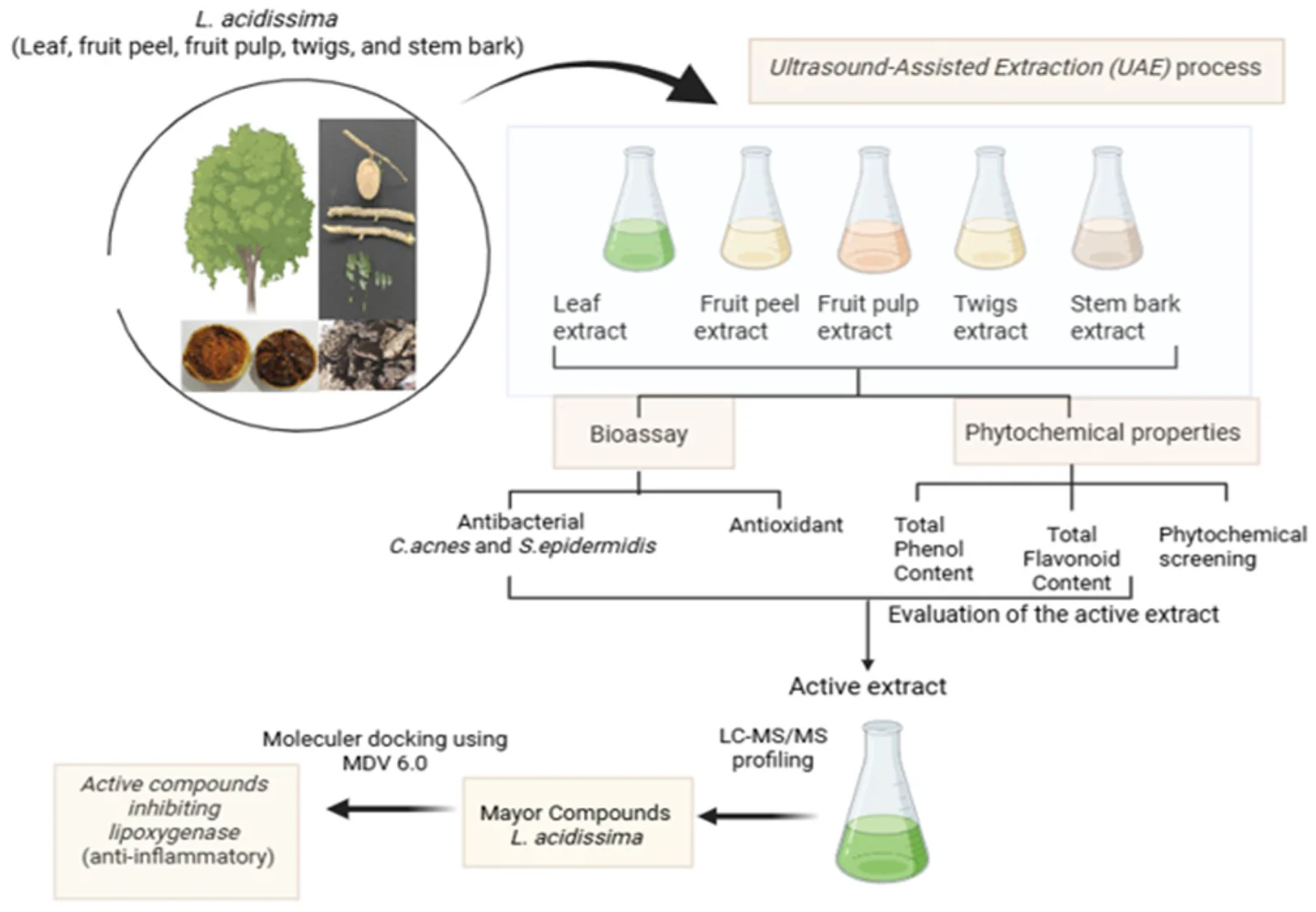
Research flowchart (Created in BioRender.com).

This study comprehensively explored the potential of various parts of *L. acidissima* through experimental and computational approaches. The study involved the preparation of extracts, phytochemical profiling, evaluation of antibacterial activity against *C. acnes* and *S. epidermidis*, antioxidant activity, and flavonoid and phenolic content. The most active extract was selected using TOPSIS based on the results of biological and phytochemical evaluations, followed by the identification of its constituent compounds. Furthermore, an in silico approach using molecular docking was employed to evaluate the potential anti-inflammatory activity of the key compounds through the inhibition of the 15-LOX enzyme. The overall research workflow used to achieve the objectives of this study is presented in Figure 4 [53].

### 3.1. Chemicals and Reagents

All solvents and chemicals used in this study were of analytical or liquid chromatography-mass spectrometry (LC-MS) grade, except where mentioned differently. Technical grade of ethanol (96%) used for the extraction process was purchased from Sigma Aldrich (St. Louis, MO, USA). Standard compounds for phytochemical quantification, including gallic acid and quercetin, were obtained from Sigma Aldrich (St. Louis, MO, USA). Reagents utilized for the antioxidant assays, namely 2,2-diphenyl-1-picrylhydrazyl (DPPH), 2,2′-azinobis-(3-ethylbenzothiazoline-6-sulfonic acid) (ABTS), and ascorbic acid were procured from Merck (Darmstadt, Germany).

Other analytical grade laboratory reagents, including sodium hydroxide (NaOH), sodium carbonate (Na_2_CO_3_), sodium acetate (CH_3_COONa), hydrochloric acid (HCl), and dimethyl sulfoxide (DMSO), were sourced from Merck (Darmstadt, Germany). The LC-MS grade solvents used for chromatographic profiling, including acetonitrile, ammonium formate, and formic acid, were supplied by Sigma Aldrich (St. Louis, MO, USA). Distilled water and water for injection were obtained locally in Indonesia.

For the microbiological assays, Mueller–Hinton agar (MHA) was purchased from Merck (Darmstadt, Germany). The bacterial strains evaluated in this study, *C. acnes* ATCC-6919 and *S. epidermidis* ATCC-12228, were provided by Microbiology Laboratory, Faculty of Pharmacy, Universitas Indonesia. Clindamycin discs used as the positive antibacterial control were obtained from Oxoid (Thermo Fisher Scientific, Waltham, MA, USA).

### 3.2. Preparation of Plant Materials and Extraction

All plant materials, including leaves, stem barks, twigs, fruit pulp, and fruit peel of *L. acidissima* were collected from a plantation in Karawang, West Java, Indonesia. The Department of Biology, Faculty of Mathematics and Natural Sciences, Universitas Indonesia, verified the plant’s taxonomic identity, and a specimen was deposited at the Depokensis Herbarium (UIDEP) specimen number 057/UN2.F3.11/PDP.02.00/2026. All samples were cleaned to remove impurities, weighed, and washed under running water. The fruit pulp was separated by scraping, whereas the fruit peel was crushed to facilitate drying. The leaves and stem bark were cut into smaller pieces, and the twigs were chopped to improve drying efficiency. All materials were dried in a hot-air oven at 40 °C, ground into powder, and sifted through a 60 mesh sieve. Before analysis, the powdered materials were weighed and stored in an airtight container.

Bioactive compounds were extracted from the powdered plant materials using the UAE method. Each plant part was extracted with 96% ethanol at a solid-to-solvent ratio of 1:10 (*w*/*v*) for 40 min at 50 °C [54]. The extracts were filtered through filter paper, and the resulting filtrates were concentrated using a rotary vacuum evaporator (BÜCHI Labortechnik AG, Flawil, Switzerland), followed by a water bath, until concentrated crude extracts were obtained. The extracts were then weighed and stored for subsequent analyses.

### 3.3. Phytochemical Screening, Total Phenol (TPC) and Total Flavonoid Content (TFC)

Preliminary phytochemical screening was conducted to identify secondary metabolites, including tannins, alkaloids, flavonoids, and saponins, using standard colorimetric reagents. The Folin–Ciocalteu method was used to calculate the total phenolic content using gallic acid as the reference standard. The extract solution was prepared by dissolving 50 mg of extract in methanol and adjusting the volume to 10 mL, yielding a concentration of 5000 μg/mL. Serial dilutions were subsequently prepared from the stock solution. Gallic acid standard solutions were prepared at various concentrations. Aliquots of either the sample or standard solution were mixed with 100 μL of 7.5% Folin–Ciocalteu reagent and incubated. Subsequently, 80 μL of 1% NaOH solution was added, and the mixture was incubated in the dark for 60 min. Absorbance was measured at 730 nm and 25 °C using a microplate reader (Molecular Devices, LLC, San Jose, USA). The gallic acid calibration curve was used to determine the total phenolic content, which was then reported as milligrams of gallic acid equivalents per gram of extract (mg GAE/g extract).

TFC was determined using the aluminum chloride colorimetric method in a microplate format, with quercetin as the reference standard. The extract solution was prepared by dissolving 500 mg of extract in 10 mL of ethanol, yielding a concentration of 50,000 μg/mL. Serial dilutions were then prepared from the stock solution. Quercetin standard solutions were prepared at various concentrations. Aliquots of either the sample or standard solution were mixed with 20 μL of 1 M sodium acetate (CH_3_COONa), 20 μL of 10% AlCl_3_, and 140 μL of distilled water, followed by incubation at 25 °C for 30 min. Using a microplate reader, absorbance was determined at 415 nm. Ethanol (96%) was used as the blank. The quercetin calibration curve was used to quantify the total flavonoid concentration, which was then reported as milligrams of quercetin equivalents per gram of extract (mg QE/g extract).

### 3.4. Antibacterial Activity

Antibacterial activity of the extracts was evaluated using the disc diffusion (Kirby-Bauer) method. Before testing, cultures of *C. acnes* (ATCC 6919) and *S. epidermidis* (ATCC12228) were reactivated on agar slants and incubated at 37 °C for 24 h. Concentrations of 3.125%, 6.25%, 12.5%, 25%, 50%, and 75% were used to prepare the test solutions. Antibacterial activity was determined using the disc diffusion method on MHA according to the procedure described by Syafriana et al. [55]. A bacterial suspension (0.5 McFarland standard) was inoculated onto Mueller-Hinton Agar (MHA) media, and sterile discs containing 20 µL of the extract were subsequently placed on the surface of the medium. Clindamycin and 10% DMSO were used as positive and negative controls, respectively. After incubation at 37 °C for 24 h, the diameters of the inhibition zones were measured and expressed in millimeters (mm). All assays were performed in triplicate (n = 3).

### 3.5. Antioxidant Activity

The DPPH and ABTS assays were used to assess the extracts’ antioxidant properties. The DPPH assay was measured spectrophotometrically, whereas the ABTS assay was performed using a microplate reader. DPPH solution (10 mg/100 mL methanol) was prepared. Standard ascorbic acid solutions and extract solutions from leaves, stem bark, twigs, fruit pulp, and fruit peel were prepared from their respective stock solutions. An aliquot of 1 mL of either the sample or standard solution was mixed with 1 mL of DPPH solution, and the volume was adjusted to 5 mL with ethanol. The absorbance at 517 nm was measured after the reaction mixture was incubated for 30 min at room temperature in the dark.

The ABTS assay was performed according to the method described by Nur et al. [56]. The ABTS radical solution was prepared by mixing 7 mM ABTS and 2.45 mM potassium persulfate (K_2_S_2_O_8_) solutions at a ratio of 1:1 (*v*/*v*), followed by incubation in the dark at room temperature for 12–16 h. The resulting radical solution was diluted with 96% ethanol to a final volume of 100 mL before use. The 7 mM ABTS solution was prepared by dissolving 96.02 mg of ABTS in distilled water and adjusting the volume to 25 mL, whereas the 2.45 mM K_2_S_2_O_8_ solution was prepared by dissolving 16.56 mg of K_2_S_2_O_8_ in distilled water and adjusting the volume to 25 mL.

Methanol (p.a.) was used to dissolve 50 mg of ascorbic acid to create the standard solution and adjust the volume to 50 mL to obtain a concentration of 1000 μg/mL, followed by serial dilution to prepare the required concentrations. The extract solution was prepared by dissolving 50 mg of extract in 96% ethanol and adjusting the volume to 10 mL to obtain a concentration of 5000 μg/mL, followed by serial dilution.

The experiment was conducted in a 96-well microplate by combining 190 μL of ABTS radical solution with 10 μL of sample or reference solution. Using a microplate reader, absorbance was measured at 734 nm following a 15 min incubation period at 25 °C in the dark. A mixture of 96% ethanol and ABTS solution was used as the blank.

The percentage inhibition of DPPH and ABTS radicals was calculated using the following equation:

(1) $$\%\;\mathrm{Inhibition}=\frac{\mathrm{Blank}\;\mathrm{Absorbance}-\mathrm{Sample}\;\mathrm{Absorbance}}{\mathrm{Blank}\;\mathrm{Absorbance}}\times 100\%$$

Antioxidant activity was represented by the IC_50_ value, calculated from the linear regression equation between the percentage inhibition and the sample concentration using the following equation:

(2) $${IC}_{50}=\frac{50-a}{b}$$

### 3.6. Multi-Criteria Decision Analysis Using TOPSIS

To identify the most optimal plant part for anti-acne applications, TOPSIS analysis was employed. This multi-criteria decision-making method integrates biological and phytochemical parameters to rank the tested extracts with equal weighting and modified TOPSIS [57,58]. In the TOPSIS analysis, the IC_50_ values obtained from the DPPH and ABTS assays were treated as minimize criteria because lower IC_50_ values indicate stronger antioxidant activity. In contrast, the inhibition zone diameters against *C. acnes* and *S. epidermidis*, TPC, and TFC were treated as maximize criteria because higher values reflect greater biological activity or phytochemical content [59].

The analysis was conducted through the following steps [58]:

#### 3.6.1. Making a Decision Matrix

Biological and phytochemical parameters, given equal weights, were used in TOPSIS. Due to the lack of evidence of weighting between evaluation criteria, equal weighting was applied. Each criterion was given an equal weight of 1/6 (0.1667), following a commonly used weighting approach in TOPSIS when unavailable in the literature. Epidemiological and microbiological studies have shown that *C. acnes* contributes to the facial skin microbiota of the bacterial population in acne lesions [60]. Antibacterial activity against *S. epidermidis* plays a role in intensifying acne-related inflammation [61]. Antioxidant activity, represented by IC_50_ values of DPPH and ABTS, was assessed because oxidative stress contributes to sebum oxidation and acne-related inflammation [62]. Meanwhile, TPC and TFC were used as indicators of bioactive compounds that may contribute to the observed biological activities [63].

#### 3.6.2. Normalization and Construction of the Weighted Normalized Matrix

To eliminate differences in measurement scales among the evaluated criteria, the decision matrix was normalized using the vector normalization method. The normalized value (*r*_(*ij*)_) was calculated using the following equation:

(3) $$n_{ij}=\frac{x_{ij}}{\surd \sum_{i=1}^{m}x_{ij}^{2}}$$

where $n_{ij}$ represents the value of the (*i*)-th alternative regarding the (*j*)-th criterion, while $r_{ij}$ represents the corresponding normalized value. This normalization process transforms the evaluation parameters into dimensionless values, enabling direct comparison among criteria despite differences in their measurement scales.

The resulting normalized values were subsequently used to calculate the weighted normalized matrix according to the following equation:

(4) $$v_{ij}=w_{j}\;\times \;n_{ij}$$

where $v_{ij\;}$ is the weighted normalized value of the (*i*)-th alternative with respect to the (*j*)-th criterion, $w_{j}$ is the weight assigned to the (*j*)-th criterion, and $r_{ij\;}$ is the normalized value. This step incorporates the relative importance of each criterion into the decision-making process.

#### 3.6.3. Determination of the Ideal Solution and Preference Values

The positive ideal solution (A^+^) and negative ideal solution (A^−^) were determined from the weighted normalized decision matrix based on the type of criterion (benefit or cost). Subsequently, the distances of each alternative from the positive (*D*^+^) and negative (*D*^−^) ideal solutions were calculated using the Euclidean distance method as follows:

(5) $$D_{i}^{+}=\;\sqrt{\sum {\left(vij-A^{+}\right)}^{2}}$$

(6) $$D_{i}^{-}=\sqrt{\sum {\left(vij-A^{-}\right)}^{2}}$$

The preference value, also referred to as the closeness coefficient ($CC_{i}$) was calculated using the following equation:

(7) $$CC_{i}=\frac{D_{i}^{-}}{D_{i}^{+}+D_{i}^{-}}$$

A higher ($CC_{i}$) value indicates a better alternative because it reflects greater proximity to the positive ideal solution and greater distance from the negative ideal solution.

### 3.7. LC-MS/MS

The phytochemical profile of the most active extract was characterized using LC-MS/MS. Chromatographic separation was performed on a C18 analytical column (2.1 × 100 mm, 1.7 μm particle size), maintained at 40 °C. The mobile phase consisted of water containing 5 mM ammonium formate (A) and acetonitrile containing 0.05% formic acid (B). A gradient elution program was applied over 23 min at a flow rate of 0.2 mL/min. The injection volume was 5 μL. Mass spectrometric analysis was acquired using a Waters Xevo G2-S QTof (Waters, Milford, MA, USA) equipped with an electrospray ionization (ESI) source in positive ion mode. The mass range was set from *m*/*z* 50 to 1200. The source and desolvation temperatures were maintained at 100 °C and 350 °C, respectively. The collision energy varied from 4 to 60 eV, while the cone and desolvation gas flow rates were set at 0 and 793 L/h, respectively. Mass spectra and chromatographic data were processed for dereplication and peak alignment using MassLynx version 4.1 and MS-DIAL version 5 [64]. The error mass of metabolite annotation strictly set at ±5 ppm.

### 3.8. Molecular Docking Analysis

Virtual Docker version 6.0 (Molegro ApS, Aarhus, Denmark) was used to predict the binding interactions underlying the antibacterial activities against *C. acnes* and *S. epidermidis*, as well as the antioxidant and anti-inflammatory activities of the identified compounds against their respective target proteins. The crystal structures of the *C. acnes* target protein (PDB ID: 6A9N; resolution, 2.10 Å), *S. epidermidis* target protein (PDB ID: 4M8I; resolution, 1.43 Å), antioxidant target protein (PDB ID: 4L7B; resolution, 2.41 Å), and 15-lipoxygenase (15-LOX; PDB ID: 7LAF; resolution, 2.44 Å) were obtained from the Protein Data Bank (PDB).

During protein preparation, water molecules, cofactors, and other non-essential components were removed to optimize the protein structures and binding sites for docking. The binding cavities were centered on the corresponding co-crystallized reference ligands at the following coordinates: *C. acnes* antibacterial target KAS III (x = −48.98, y = 24.24, z = 22.68), *S. epidermidis* antibacterial target FtsZ (x = −18.57, y = −9.78, z = 20.02), antioxidant target KEAP1 (x = −3.47, y = 2.29, z = −27.53), and 15-LOX (x = −50.51, y = 6.33, z = 545.58). A grid box size of 30 × 30 × 30 Å was used for all docking simulations (x, y, z), with a grid size of 30 × 30 × 30 Å [65].

The 2D structures of the candidate and reference ligands (NDGA, baicalein, zileuton, erythromycin, benzoyl peroxide, ascorbic acid, and clindamycin) were obtained from the NCBI PubChem database, converted into 3D conformations, and energy-minimized using ChemDraw Professional 20.0 (Revvity Signals Software, Inc., Waltham, MA, USA) [66]. The molecular docking protocols were validated by redocking the respective native ligand [67]. The protocols successfully reproduced the co-crystallized ligand conformations, with root mean square deviation (RMSD) values of 2.07 Å for the *C. acnes* antibacterial target, 0.49 Å for the *S. epidermidis* antibacterial target, 0.88 Å for the antioxidant target, and 1.53 Å for 15-lipoxygenase (15-LOX), all within the acceptable threshold, confirming the validity of the docking protocols. The results of ligand–protein complexes were evaluated based on the Rerank Score and hydrogen bond energy, followed by 2D visualization. Physicochemical properties and drug-likeness (Lipinski’s Rule of Five) were predicted using the SwissADsME web server [68]. Docking results were evaluated based on MolDock score, Rerank score, hydrogen-bond interactions, and amino acid residues involved in ligand binding. The ligand–protein interactions were subsequently visualized in 2D formats to facilitate the interpretation of the binding modes and interaction patterns.

### 3.9. Statistical Analysis

The results of the biological assays are presented as the mean ± standard error of the mean (SEM). Prior to hypothesis testing, the normal distribution of the data and homogeneity of variances were verified using the Shapiro–Wilk and Levene’s tests. Statistical evaluation was performed via one-way analysis of variance (ANOVA) followed by Tukey’s post hoc test using GraphPad Prism version 10.0 (GraphPad Software, San Diego, CA, USA). A value of *p* < 0.05 was considered statistically significant. Subsequent extract ranking was executed through TOPSIS based on the biological and phytochemical parameters.

## 4. Conclusions

This study demonstrates that the morphological parts of *L. acidissima* exhibit distinct antibacterial and antioxidant profiles. Among the plant parts evaluated, the leaf extract exhibited the most potent overall biological activities, including activity against bacterial colonization, oxidative stress, and inflammation. This result supports its potential as a multitarget anti-acne candidate. The integration of in vitro and in silico findings provides a preclinical rationale for the further development of the leaf extract as a topical anti-acne agent. However, the predicted 15-LOX inhibitory potential requires experimental validation. Future studies should prioritize the isolation and structural characterization of the major bioactive constituents, followed by evaluation of the purified compounds using enzyme-based assays and in vivo models to confirm their biological activities, skin delivery, safety, and therapeutic relevance.

## Figures and Tables

**Table 1:** Phytochemical contents and extraction yield of *Limonia acidissima*.

No.	Plant Parts	The Presence of Phytoconstituents				Yield (%)
		Alkaloid	Flavonoid	Tanin	Saponin	
1	Fruit pulp	+	+	+	+	25.58
2	Stem bark	+	+	+	+	13.60
3	Leaves	+	+	+	+	13.50
4	Fruit peel	+	+	+	+	9.80
5	Twigs	+	+	+	+	7.02

**Table 2:** Total phenolic content and total flavonoid content of *Limonia acidissima*.

No.	Plant Parts	Total Phenol Content (mg GAE/g Extract) ± SEM	Total Flavonoid Content (mg QE/g Extract) ± SEM
1	Fruit pulp	8.80 ± 0.14 ^a^	25.33 ± 0.14 ^a^
2	Stem bark	39.95 ± 1.14 ^b^	16.37 ± 0.06 ^b^
3	Leaves	66.11 ± 0.68 ^c^	197.36 ± 3.06 ^c^
4	Fruit peel	61.58 ± 1.75 ^c^	13.65 ± 1.06 ^b^
5	Twigs	27.22 ± 0.43 ^d^	17.24 ± 0.59 ^b^

Data for TPC and TFC are reported as mean ± standard error of the mean (SEM) from three replicate analyses. Means bearing different superscript letters are significantly different according to one-way ANOVA and Tukey’s post hoc test (*p* < 0.05, *n* = 3).

**Table 3:** Mean inhibition zone diameter of *Limonia acidissima* against *Cutibacterium acnes* (disc diffusion assay).

Concentration (%)	Inhibition Zone Diameter (mm) ± SEM				
	Leaves	Fruit Peel	Fruit Pulp	Stem Bark	Twigs
Clindamycin	21.24 ± 0.80 ^a^	22.12 ± 1.13 ^a^	22.52 ± 0.25 ^a^	22.60 ± 2.00 ^a^	23.02 ± 0.47 ^a^
75	8.37 ± 0.39 ^b^	13.14 ± 3.85 ^b^	9.52 ± 0.33 ^b^	10.73 ± 1.05 ^b^	11.70 ± 0.79 ^b^
50	7.76 ± 0.28 ^b^	9.60 ± 1.41 ^b^	7.56 ± 0.60 ^c^	9.74 ± 1.36 ^b^	7.44 ± 0.12 ^c^
25	7.34 ± 0.18 ^b^	7.53 ± 0.27 ^b^	7.55 ± 0.43 ^c^	9.74 ± 1.32 ^b^	7.42 ± 0.10 ^c^
12.50	7.25 ± 0.07 ^b^	7.19 ± 0.17 ^b^	7.50 ± 0.30 ^c^	9.58 ± 1.39 ^b^	7.21 ± 0.09 ^c^
6.25	6.74 ± 0.33 ^b^	7.18 ± 0.15 ^b^	7.14 ± 0.38 ^c^	9.53 ± 1.41 ^b^	6.92 ± 0.04 ^c^
3.125	6.38 ± 0.33 ^b^	6.28 ± 0.07 ^b^	6.48 ± 0.22 ^c^	9.10 ± 1.76 ^b^	6.62 ± 0.18 ^c^
10% DMSO	0.00 ± 0.00	0.00 ± 0.00	0.00 ± 0.00	0.00 ± 0.00	0.00 ± 0.00

Data are presented as the mean ± standard error of the mean (SEM) from three replicates. Superscript letters that differ denote statistically significant differences among the tested extracts (one-way ANOVA followed by Tukey’s post hoc analysis, *p* < 0.05, *n* = 3).

**Table 4:** Mean inhibition zone diameter of *Limonia acidissima* against *Staphylococcus epidermidis* (disc diffusion assay).

Concentration (%)	Inhibition Zone Diameter (mm) ± SEM				
	Leaves	Fruit Peel	Fruit Pulp	Stem Bark	Twigs
Clindamycin	24.23 ± 0.53 ^a^	21.32 ± 1.42 ^a^	22.99 ± 0.63 ^a^	24.29 ± 0.24 ^a^	19.03 ± 4.36 ^a^
75	12.75 ± 0.44 ^b^	16.95 ± 0.78 ^b^	15.35 ± 0.93 ^b^	12.70 ± 1.79 ^b^	14.80 ± 0.42 ^b^
50	11.91 ± 0.70 ^bc^	13.75 ± 1.17 ^bc^	11.77 ± 1.37 ^c^	11.76 ± 1.27 ^bc^	12.45 ± 0.79 ^c^
25	11.29 ± 0.61 ^bc^	10.34 ± 0.72 ^cd^	9.84 ± 0.88 ^d^	10.60 ± 1.36 ^bc^	12.03 ± 0.20 ^c^
12.50	10.80 ± 0.30 ^bc^	9.39 ± 0.56 ^d^	9.80 ± 0.23 ^d^	10.48 ± 1.44 ^bc^	11.15 ± 0.73 ^cd^
6.25	10.39 ± 0.66 ^bc^	9.01 ± 0.54 ^d^	9.65 ± 0.46 ^d^	8.84 ± 1.04 ^c^	10.45 ± 0.44 ^d^
3.125	9.15 ± 0.71 ^c^	8.89 ± 0.25 ^d^	9.04 ± 0.44 ^d^	8.41 ± 1.09 ^c^	9.65 ± 0.23 ^d^
10% DMSO	0.00 ± 0.00	0.00 ± 0.00	0.00 ± 0.00	0.00 ± 0.00	0.00 ± 0.00

Data is presented as the mean ± standard error of the mean (SEM) of three replicates. Superscript letters that differ denote statistically significant differences among the tested extracts (one-way ANOVA followed by Tukey’s post hoc analysis, *p* < 0.05, *n* = 3).

**Table 5:** Antioxidant activity of the *Limonia acidissima* extracts.

Plant Parts	DPPH (IC_50_ μg/mL)	ABTS (IC_50_ μg/mL)
Fruit pulp	228.52 ± 35.43 ^a^	799.21 ± 17.16 ^a^
Fruit peel	219.70 ± 36.92 ^a^	88.50 ± 1.21 ^b^
Leaves	78.16 ± 13.37 ^b^	78.42 ± 0.43 ^c^
Stem bark	71.64 ± 10.00 ^b^	76.90 ± 0.69 ^c^
Twigs	50.22 ± 2.99 ^bc^	73.29 ± 0.44 ^c^
Ascorbic acid	8.32 ± 0.08 ^c^	5.72 ± 0.05 ^d^

Data is presented as the mean ± standard error of the mean (SEM) of three replicates. Superscript letters that differ denote statistically significant differences among the tested extracts (one-way ANOVA followed by Tukey’s post hoc analysis, *p* < 0.05, *n* = 3).

**Table 6:** Positive ideal solution (A^+^) and negative ideal solution (A^−^) for TOPSIS analysis.

Criteria	A^+^	A^−^
DPPH	0.02473	0.11253
ABTS	0.01500	0.16359
*C. acnes*	0.09616	0.06636
*S. epidermidis*	0.07959	0.06936
TPC	0.10714	0.01426
TFC	0.16376	0.01133

**Table 7:** TOPSIS analysis results of different parts of *Limonia acidissima*.

Criteria	*D* ^+^	*D* ^−^	*CC_i_*	Ranking
Leaf	0.03215	0.24321	0.88325	1
Twig	0.16220	0.17799	0.52321	2
Stem bark	0.15891	0.17434	0.52314	3
Fruit peel	0.17661	0.16886	0.48878	4
Fruit pulp	0.24409	0.01120	0.04387	5

**Table 8:** Compounds detected in the leaf extract of *Limonia acidissima*.

No	Compound	Formula	Ion Mass (*m*/*z*)	Adduct Type	Area Under Curve	Retention Time (min)	Classes
1	Indole	C_8_H_7_N	118.067	[M+Na]^+^	30,470	4	Alkaloid
2	Caffeate	C_9_H_8_O_4_	163.039	[M+H]^+^	14,572	4.09	phenolic
3	Cyanidin-3-O-galactoside	C_21_H_21_O_11_^+^	449.104	[M+H]^+^	10,217	4.57	Flavonoid
4	Vitexin	C_21_H_20_O_10_	433.113	[M+H]^+^	8,731,273	4.92	Flavonoid
5	Quercetin	C_15_H_10_O_7_	303.05	[M+K]^+^	161,461	5.01	Flavonoid
6	Methyleugenol	C_11_H_14_O_2_	179.107	[M+H]^+^	187,435	6.06	Phenolic
7	Linolenic acid	C_18_H_30_O_2_	279.2311	[M+H]^+^	21,337	12.75	Lipid

**Table 9:** Molecular docking analysis of test compounds and reference ligands.

***C. acnes*** **KAS III Target PDB ID: 6A9N**				
**Compound**	**MolDock Score**	**Rerank Score**	**H-Bond Energy**	**Interaction Residues**
Clindamycin *	−95.199	−79.224	−9.862	Ile136, Asn142, Gly 139, His10, Pro8, Arg137
Linolenic acid	−103.654	−78.470	−2.5	Asp180, His10, Ser179, Pro8
NDGA *	−93.842	−77.578	−13.161	Asp180, His10, Ala178, Gly11, Tyr12, Ser179, Gly139, Asn142, Arg7
Quercetin	−87.524	−74.807	−11.775	Asn142, Gly139, His10, Ala178, Pro8, Ser179, Ile136, Tyr12
Vitexin	−80.948	−69.562	−7.906	Arg137, Ile136, Pro8, His10, Asn142, Tyr143, Asp180, Ser179
Benzoyl peroxide *	−75.290	−67.302	−0.605	His10, Arg137, Gly139, Pro8
Caffeate	−76.454	−63.329	−9.812	Gly11, Tyr12, Ser179, Asp180, Ala178, His10, Arg137, Ile136
Baicalin *	−73.600	−63.040	−9.933	Pro8, Asp180, Ala178, Tyr12, His10, Ile136, Ser179
Cyanidin-3-O-galactoside	−94.173	−54.933	−16.148	Tyr143, Asn142, Ala178, Pro8, Asp180, Ser179, His10
Methyleugenol	−66.471	−54.920	0	Pro8, Tyr12, His10, Ala178, Ile136
Indole	−48.402	−39.202	0	His10, Tyr12
Erythromycin *	−93.080	−34.251	−7.132	His10, Ala178, Asp180, Pro8, Arg7, Asn142
***S. epidermidis*** **FtsZ Target PDB ID: 4M8I**				
**Compound**	**MolDock Score**	**Rerank Score**	**H-Bond Energy**	**Interaction Residues**
Cyanidin-3-O-galactoside	−129.128	−109.588	−11.166	Glu139, Arg143, Thr133, Met105, Gly22, Ala103, Asp187, Arg29, Phe136, Gly104
NDGA *	−119.341	−105.11	−13.477	Arg143, Val131, Ala103, Thr102, Met105, Glu139, Gly107
Vitexin	−118.529	−99.83	−18.424	Gly104, Gly107, Met105, Asn166, Glu139, Asp187, Gly22, Asn25, Arg29
Linolenic acid	−117.704	−98.356	−6.431	Gly21, Gly104, Gly108, Gly110, Thr109
Quercetin	−109.763	−97.154	−11.840	Met105, Gly104, Thr133, Ala103, Thr102, Gly22, Arg29, Asn166, Arg143, Gly107
Clindamycin *	−111.999	−96.592	−13.588	Asn166, Glu139, Arg134, Pro135, Thr133, Gly107, Gly106, Met105, Gly110, Gly104, Ala73, Thr109, Gly21
Baicalin *	−104.872	−91.902	−13.086	Gly104, Thr111, Gly107, Ala71, Thr109, Gly72, Ala73, Arg143, Met105, Gly110
Benzoyl peroxide *	−99.827	−86.380	−0.394	Ala26, Thr102, Arg29, Phe183, Ile164
Caffeate	−92.418	−77.798	−12.123	Arg29, Phe183, Asp187, Gly22, Thr102, Val131, Leu190, Ala186
Methyleugenol	−82.502	−70.467	−0.466	Arg29, Ala186
Erythromycin *	−110.492	−61.676	−12.471	Glu139, Thr133, Met105, Arg143, Asn25, Asp46, Gly107, Gly104, Gly108, Thr109, Gly110
Indole	−60.962	−51.726	0	Thr133
**Antioxidant Target KEAP1 PDB ID: 4L7B**				
**Compound**	**MolDock Score**	**Rerank Score**	**H-Bond Energy**	**Interaction Residues**
Cyanidin-3-O-galactoside	−149.732	−132.253	−13.507	Asn414, Ser363, Gly364, Val604, Leu365, Gly603, Leu557, Gly509, Ser508, Arg415
NDGA *	−120.383	−102.962	−9.226	Ala510, Gly511, Gly509, Leu557, Arg415, Ser508
Quercetin	−114.784	−99.745	−10.978	Gly462, Leu557, Gly509, Leu365, Ile416, Gly364, Arg415, Ser555, Ser508,
Linolenic acid	−126.48	−98.553	−1.858	Gly364, Ser555
Vitexin	−116.215	−93.461	−5.419	Arg483, Phe478, Arg415, Ser508, Ile461, Gly462, Gly509, Gly364, Ser602, Ser555
Baicalin *	−104.312	−91.459	−7.656	Gly462, Leu365, Val604, Leu557, Gly509
Caffeate	−82.019	−69.308	−7.439	Leu557, Gly509, Val463, Ala510, Arg415
Methyleugenol	−80.341	−68.091	0	-
Ascorbic acid *	−75.359	−66.587	−10.311	Ala510, Gly364, Leu365, Val463, Ile416, Gly462, Leu557
Indole	−61.497	−52.242	−6866	Gly462, Arg483, Ser508
**Anti-Inflammatory Target 15-LOX PDB ID: 7LAF**				
**Compound**	**MolDock Score**	**Rerank Score**	**H-Bond Energy**	**Interaction Residues**
Linolenic acid	−121.867	−101.025	0	Val426
NDGA *	−119.419	−98.890	−5	His378, Glu369, Leu420, Leu419
Baicalin *	−97.881	−79.835	−4.829	His378, Leu374, Glu369, Leu420
Zileuton *	−98.586	−76.540	−5.374	Ala416, Glu369, Ile676, Ile412, His378
Caffeate	−82.197	−69.890	−4.820	Ile676, Glu369, Ala416
Quercetin	−86.703	−69.707	−4.886	Ile421, Leu420, Leu374, His373, Glu369, His378, Leu415, Leu419, Ile412
Methyleugenol	−79.537	−67.932	0	Glu369
Vitexin	−121.293	−62.943	−0.891	Arg417, Leu374, Asn413, His378, His 373, Glu369, Leu420, ala416, Ile412, Ile421
Indole	−57.798	−48.478	0	-
Clindamycin *	−111.713	−42.009	−1.863	Leu610, Leu607, Phe438, Leu420, Glu 369, Leu374, His373, Ile676, Ile412, Ala606
Cyanidin-3-O-galactoside	−98.892	6.865	−11.345	Ala416, Glu369, Ile676, His373, Phe438, Leu420, Leu374, Leu379, Arg417, Ile421, His378, Asn413, Ile412

* Reference ligand.

**Table 10:** Lipinski’s rule of five and physicochemical properties of major compounds predicted by SwissADME.

Compound	Molecular Weight (Da) < 500	Log P ≤ 5	Hydrogen Bond Donor (HBD) ≤ 5	Hydrogen Bond Acceptor (HBA) ≤ 10	Lipinski Violation ≤ 1	Topological Polar Surface Area (TPSA)	Water Solubility (ESOL)	Skin Permeation (Log Kp)
Linolenic acid	264.40	5.12	1	2	Yes	37.30	−4.42	−3.71
Caffeate	180.16	0.92	3	4	Yes	77.76	−1.89	−6.58
Quercetin	302.24	0.79	5	7	Yes	131.36	−3.19	−7.01
Methyleugenol	178.23	2.56	0	2	Yes	18.46	−2.61	−5.60
Vitexin	432.38	−0.97	7	10	Yes	181.05	−2.34	−9.35
Indole	117.15	2.03	1	0	Yes	15.79	−2.60	−5.56
Cyanidin-3-O-galactoside	449.38	−1.18	8	11	No	193.44	−1.59	−10.33

## Data Availability

The original contributions presented in this study are included in the article/Supplementary Materials. Further inquiries can be directed to the corresponding author.

## Supplementary Materials

The following supporting information can be downloaded at https://www.mdpi.com/article/10.3390/molecules31183234/s1. Figure S1: Inhibition zones of *L. acidissima* extracts against *C. acnes*: (A) leaf, (B) fruit peel, (C) fruit pulp, (D) stem bark, and (E) twig; and against *S. epidermidis*: (F) leaf, (G) fruit peel, (H) fruit pulp, (I) stem bark, and (J) twig; Table S1: Compounds identified by LC-MS/MS. Figure S2: Two-dimensional interaction maps of the ligand–receptor complexes against *C. acnes*: (A) Linolenic acid, (B) NDGA, (C) Baicalin, (D) Caffeate, (E) Quercetin, (F) Methyleugenol, (G) Vitexin, (H) Indole, (I) Clindamycin, (J) Cyanidin-3-O-galactoside, (K) Erythromycin, and (L) Benzoyl peroxide. Blue dashed lines indicate hydrogen bonds, whereas red dashed lines represent steric (van der Waals) interactions. Figure S3: Two-dimensional interaction maps of the ligand–receptor complexes against *S. epidermidis*: (A) Linolenic acid, (B) NDGA, (C) Baicalin, (D) Caffeate, (E) Quercetin, (F) Methyleugenol, (G) Vitexin, (H) Indole, (I) Clindamycin, (J) Cyanidin-3-O-galactoside, (K) Erythromycin, and (L) Benzoyl peroxide. Blue dashed lines indicate hydrogen bonds, whereas red dashed lines represent steric (van der Waals) interactions. Figure S4: Two-dimensional interaction maps of the ligand–receptor complexes against antioxidants: (A) Linolenic acid, (B) Baicalin, (C) NDGA, (D) Caffeate, (E) Quercetin, (F) Methyleugenol, (G) Vitexin, (H) Indole, (I) Cyanidin-3-O-galactoside, and (J) Ascorbic acid. Blue dashed lines indicate hydrogen bonds, whereas red dashed lines represent steric (van der Waals) interactions. Figure S5: Two-dimensional interaction maps of ligand and receptor complexes against 15-LOX: (A) Linolenic acid, (B) NDGA, (C) Baicalin, (D) Caffeate, (E) Zileuton, (F) Quercetin, (G) Methyleugenol, (H) Vitexin, (I) Indole, (J) Clindamycin, (K) Cyanidin-3-O-galactoside. Blue dashed lines indicate hydrogen bonds, whereas red dashed lines represent steric (van der Waals) interactions.

## Abbreviations

ABTS	2,2′-Azino-bis(3-ethylbenzothiazoline-6-sulfonic acid)
ADME	Absorption, distribution, metabolism, and excretion
DPPH	2,2-diphenyl-1-picrylhydrazyl
EPS	Extracellular polymeric substances
ESOL	Estimated Solubility
GAE	Gallic acid equivalent
HBA	Hydrogen Bond Acceptor
HBD	Hydrogen Bond Donor
IL-1β, IL-6, and IL-8	Interleukin-1 beta, Interleukin 6, and Interleukin 8
LC-MS/MS	Liquid Chromatography-Tandem Mass Spectrometry
MHA	Mueller–Hinton Agar
MAPK	Mitogen-activated protein kinase
NCBI	National Center for Biotechnology Information
NDGA	Nordihydroguaiaretic Acid
NF-κB	Nuclear Factor Kappa B
PDB	Protein Data Bank
QE	Quercetin Equivalent
ROS	Reactive oxygen species
SMILES	Simplified Molecular Input Line Entry System
TFC	Total flavonoid content
TLR2	Toll-like Receptor 2
TNF-α	Tumor Necrosis Factor-alpha
TOPSIS	Technique for Order Preference by Similarity to the Ideal Solution
TPC	Total phenolic content
TPSA	Topological Polar Surface Area
UAE	Ultrasound-Assisted Extraction
15-LOX	15-Lipoxygenase
