# Supplementary material for: Bioassay-Guided Evaluation and LC-MS/MS Characterization of Five Limonia acidissima L. Plant Parts: A Multitarget Approach Against Acne Vulgaris
Source: Molecules. 2026 Sep 13;31(18):3234. doi: 10.3390/molecules31183234 (PMC13609078; doi:10.3390/molecules31183234)
Supplement: Supplementary file 1 [file molecules-31-03234-s001.zip › molecules-4491699-supplementary.pdf]

Supplementary Material

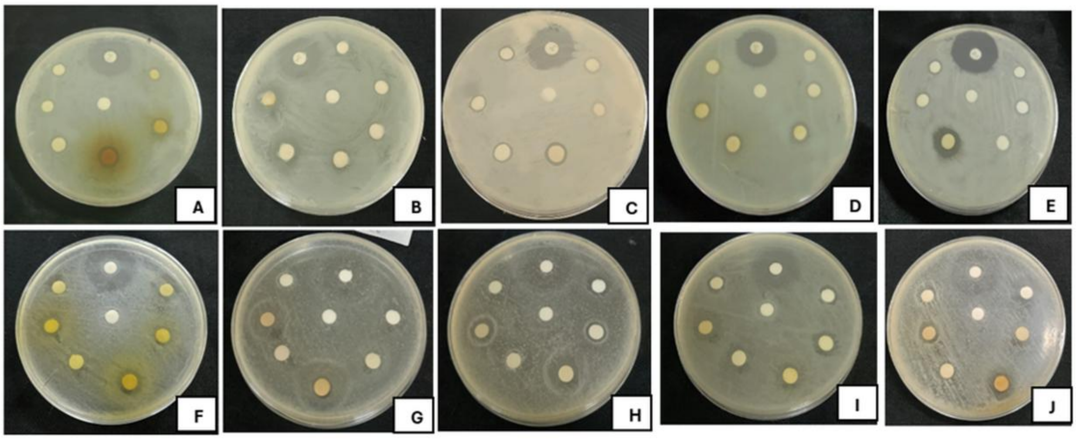

Supplementary Figure S1. Inhibition zones of *L. acidissima* extracts against *C. acnes*: (A) leaf, (B) fruit peel, (C) fruit pulp, (D) stem bark, and (E) twig; and against *S. epidermidis*: (F) leaf, (G) fruit peel, (H) fruit pulp, (I) stem bark, and (J) twig

Supplementary Table S1. Compounds identified by LC-MS/MS

No		**Formula**	**Metabolite group**
1	Argininosuccinic acid	$C_{10}H_{18}N_4O_6$	Amino acid derivative/ peptide
2	Indole-3-Acetaldehyde	$C_{10}H_9NO$	Alkaloid (Indole)
3	6,8-Diprenylorobol	$C_{25}H_{28}O_6$	Prenylated flavonoid (Isoflavonoids)
4	Shearinine	$C_{37}H_{45}NO_6$	Alkaloid (Indole)
5	Skimmin	$C_{15}H_{16}O_8$	Phenolic
6	(2R)-6-methylpiperidine-2-carboxylic acid	$C_7H_{13}NO_2$	Alkaloid / Heterocyclic Base
7	5-hydroxy-4-methoxy-5-prop-1-en-2-ylfuran-2-one	$C_8H_{10}O_4$	Other secondary metabolites
8	L-Sinoacutine	$C_{19}H_{21}NO_4$	Alkaloid / Heterocyclic Base
9	Methyl 3-aminopyrazine-2-carboxylic acid	$C_6H_7N_3O_2$	Alkaloid / Heterocyclic Base
10	4-methoxy-9H-xanthen-9-one	$C_{14}H_{10}O_3$	Phenolic
11	Dehydrotumulosic acid	$C_{31}H_{48}O_4$	Triterpenoid
12	Quercetin	$C_{15}H_{10}O_7$	Flavonoid
13	Tetrahydrocoptisine	$C_{19}H_{17}NO_4$	Alkaloid
14	Cyanidin-3-O-galactoside	$C_{21}H_{21}O_{11}$	Flavonoid Glikosida
15	Kaempferol	$C_{15}H_{10}O_6$	Flavonoid
16	4-Aminobenzoate	$C_7H_7NO_2$	Amino Acids / Peptides
17	3,4-Dimethoxybenzaldehyde	$C_9H_{10}O_3$	Phenolic
18	Theophylline	$C_7H_8N_4O_2$	Alkaloid / Heterocyclic Base
19	Indole	C_8H_7N	Alkaloid (Indole)
20	Androsterone	$C_{19}H_{30}O_2$	Steroid

21	Cyclopamine	$C_{27}H_{41}NO_2$	Alkaloid
22	Ganodermanondiol	$C_{30}H_{48}O_3$	Triterpenoid
23	Kaempferol 3-O-rutinoside	$C_{27}H_{30}O_{15}$	Flavonoid glycoside
24	5-Methoxytryptophol	$C_{11}H_{13}NO_2$	Alkaloid (Indole)
25	Villol	$C_{23}H_{22}O_9$	Flavonoid
26	Glomellic acid	$C_{25}H_{28}O_9$	Polyketide
27	beta-naphthoflavone	$C_{19}H_{12}O_2$	Flavonoid
28	Haematoidin	$C_{33}H_{36}N_4O_6$	Heterocyclic Base
29	Ketoisovaleric acid	$C_5H_8O_3$	Aliphatic organic acid
30	Shikimic acid	$C_7H_{10}O_5$	Organic acid
31	6-hydroxybenzofuran-3(2H)-one	$C_8H_6O_3$	Phenolic
32	Soyasapogenol A	$C_{30}H_{50}O_4$	Triterpenoid
33	2-Methylindole	C_9H_9N	Alkaloid (Indole)
34	Delphinidin 3-galactoside	$C_{21}H_{21}O_{12}$	Flavonoid glycoside
35	Fallacinal	$C_{16}H_{10}O_6$	Phenolic
36	Theobromine	$C_7H_8N_4O_2$	Alkaloid
37	Soyasapogenol B	$C_{30}H_{50}O_3$	Triterpenoid
38	Shanzhiside	$C_{17}H_{26}O_{11}$	Glycoside
39	Linolenic acid	$C_{18}H_{30}O_2$	Fatty acid
40	Xanthohumol	$C_{21}H_{22}O_5$	Prenylated flavonoid
41	6-Demethoxytangeretin	$C_{19}H_{22}NO_4$	Flavonoid
42	Rhodinyl Acetate	$C_{12}H_{22}O_2$	Terpenoid
43	Beta-Caryophyllene Alcohol	$C_{15}H_{26}O$	Seskuiterpenoid
44	Lasiocarpine	$C_{32}H_{45}NO8$	Alkaloid
45	Tsugaric acid A	$C_{32}H_{50}O_4$	Triterpenoid
46	Bufotenine	$C_{12}H_{16}N_2O$	Alkaloid (Indole)
47	glycyrrhetic acid	$C_{30}H_{46}O_4$	Triterpenoid
48	Diallyl Sulfide	$C_6H_{10}S$	Senyawa Organosulfur
49	Wilforlide A	$C_{30}H_{46}O_3$	Triterpenoid
50	Lobariolcarboxylic acid (Lobarin)	$C_{25}H_{30}O_9$	Phenolic
51	Ranaconitine	$C_{32}H_{44}N_2O_9$	Alkaloid / Heterocyclic Base
52	3-Indoleacetic acid	$C_{10}H_9NO_2$	Alkaloid (Indole)
53	Dehydronuciferin	$C_{19}H_{19}NO_2$	Alkaloid / Heterocyclic Base
54	Liensinine Diperchlorate	$C_{37}H_{43}ClN_2O_{10}$	Alkaloid / Heterocyclic Base
55	Casticin	$C_{23}H_{26}N_2O_4$	Alkaloid / Heterocyclic Base
56	Austrobuxusin E	$C_{27}H_{40}O_7$	Other Secondary Metabolites
57	Dehydropachymic acid	$C_{33}H_{50}O_5$	Triterpenoid
58	Sekikaic acid	$C_{22}H_{26}O_8$	Flavonoid
59	6-Shogaol	$C_{17}H_{24}O_3$	Phenolic
60	Alpha Cyperone	$C_{15}H_{22}O$	Seskuiterpenoid
61	Licoflavone A	$C_{15}H_{10}O_8$	Flavonoid

62	ursolic acid	$C_{30}H_{48}O_3$	Triterpenoid
63	Stigmasta-4,22-dien-3-one	$C_{27}H_{39}NO_2$	Alkaloid / Heterocyclic Base
64	7-O-Methyl morroniside	$C_{21}H_{18}O_9$	Flavonoid Glikosida
65	Phenylpropanolamine	$C_9H_{13}NO$	Compounds Containing Nitrogen (Amines/Amides)
66	Hyoscyamine	$C_{17}H_{23}NO_3$	Alkaloid / Heterocyclic Base
67	Corosolic acid	$C_{30}H_{48}O_4$	Triterpenoid
68	Syringic acid	$C_9H_{10}O_5$	Phenolic Acid
69	gomisin A	$C_{27}H_{41}NO_2$	Alkaloid / Heterocyclic Base
70	Carylophyllene Oxide	$C_{14}H_{22}O$	Other Secondary Metabolites
71	Daphnoretin	$C_{21}H_{34}O_4$	Phenolic
72	Echinocystic acid	$C_{30}H_{48}O_4$	Triterpenoid
73	Hordenine	$C_{10}H_{15}NO$	Compounds Containing Nitrogen (Amines/Amides)
74	Indole-3-propionic acid	$C_{11}H_{11}NO_2$	Alkaloid (Indole)
75	reserpinine	$C_{21}H_{22}O_6$	Flavonoid
76	Vitexin	$C_{27}H_{39}NO_2$	Alkaloid / Heterocyclic Base
77	mahanimbine	$C_{19}H_{21}NO_4$	Alkaloid / Heterocyclic Base
78	Raddeanin A	$C_{47}H_{76}O_{16}$	Triterpenoid
79	daniellic acid	$C_{20}H_{34}O$	Diterpenoid
80	Loliolide	$C_{11}H_{16}O_3$	Other Secondary Metabolites
81	gitoxigenin	$C_{19}H_{18}O_9$	Flavonoid
82	Gramine	$C_{11}H_{14}N_2$	Alkaloid (Indole)
83	trans-Zeatin	$C_{10}H_{13}N_5O$	Alkaloid / Heterocyclic Base
84	Echimidine	$C_{19}H_{18}O_8$	Flavonoid
85	scopolamine	$C_{15}H_{16}O_8$	Phenolic
86	Uvarigrin	$C_{37}H_{68}O_6$	Other Secondary Metabolites
87	Obtusaquinone	$C_{16}H_{14}O_3$	Simple Aromatic Compounds
88	Dehydroeburicoic acid monoacetate	$C_{33}H_{50}O_4$	Triterpenoid
89	3-Acetyl-11-keto-beta-boswellic Acid	$C_{32}H_{48}O_5$	Triterpenoid
90	methyl 3-(3,4-dihydroxy-5-phenyloxolan-2-yl)-3-hydroxypropanoate	$C_{14}H_{18}O_6$	Simple Aromatic Compounds
91	Homoveratrylamine	$C_{10}H_{15}NO_2$	Compounds Containing Nitrogen (Amines/Amides)
92	Bauerine C	$C_{12}H_8Cl_2N_2O$	Alkaloid / Heterocyclic Base
93	Benzaldehyde	C_7H_6O	Simple Aromatic Compounds
94	Pachyrrhizin	$C_{23}H_{25}NO$	Alkaloid (Indole)
95	9H-xanthen-9-one	$C_{13}H_8O_2$	Other Secondary Metabolites
96	3-Methoxycolensoic acid	$C_{26}H_{32}O_8$	Flavonoid
97	8-hydroxy-6,7-dimethoxy-2H-chromen-2-one	$C_{11}H_{10}O_5$	Phenolic
98	Peimisine	$C_{27}H_{41}NO_3$	Alkaloid / Heterocyclic Base

99	veratramine	$C_{20}H_{24}O_7$	Diterpenoid
100	friedelin	$C_{25}H_{28}O_6$	Flavonoid
101	synephrine	$C_9H_{13}NO_2$	Compounds Containing Nitrogen (Amines/Amides)
102	khellin	$C_{14}H_{12}O_5$	Phenolic
103	Lasiocarpine N-oxide	$C_{30}H_{50}O$	Triterpenoid
104	(+)-Corypalmine	$C_{20}H_{34}O_4$	Diterpenoid
105	Di-O-methylfraxetin	$C_{12}H_{12}O_5$	Phenolic
106	Dihydrochelerythrine	$C_{19}H_{21}NO_4$	Alkaloid / Heterocyclic Base
107	N-[2-(1H-Indol-3-yl)ethyl]acetamide	$C_{12}H_{14}N_2O$	Alkaloid (Indole)
108	Squamatic acid	$C_{22}H_{26}N_2O_4$	Alkaloid (Indole)
109	GUAIAZULENE	$C_{15}H_{18}$	Seskuiterpenoid
110	Kynurenic acid; CE0; HCZHHEIFKROPDY-UHFFFAOYSA-N	$C_{10}H_7NO_3$	Alkaloid / Heterocyclic Base
111	Chlorogenic acid	$C_{19}H_{12}O_7$	Flavonoid Glikosida
112	3beta-acetoxy-17(21)-hopen-1-one	$C_{32}H_{50}O_3$	Triterpenoid
113	Dihydrolycorine	$C_{16}H_{19}NO_4$	Alkaloid / Heterocyclic Base
114	Dihydro-8-deoxy-lactucin	$C_{15}H_{18}O_4$	Seskuiterpenoid
115	(2R,3R)-3,7,4'-Trihydroxy-5-methoxy-8-prenylflavanone	$C_{19}H_{22}O_5$	Flavonoid
116	(-)-Nuciferine	$C_{19}H_{21}NO_2$	Alkaloid / Heterocyclic Base
117	Huperzine A	$C_{15}H_{18}N_2O$	Alkaloid / Heterocyclic Base
118	Phenylacetaldehyde	C_8H_8O	Simple Aromatic Compounds
119	3,5,6,7,8,3',4'-Heptamethoxyflavone	$C_{22}H_{24}O_9$	Flavonoid
120	10-Gingerol	$C_{21}H_{19}NO_4$	Alkaloid / Heterocyclic Base
121	asiatic acid	$C_{30}H_{48}O_5$	Triterpenoid
122	Myricetin	$C_{20}H_{28}O_3$	Diterpenoid
123	Isovitexin	$C_{21}H_{20}O_{10}$	Flavonoid Glikosida
124	Quinolone	C_9H_7NO	Alkaloid / Heterocyclic Base
125	Ephedrine	$C_{10}H_{15}NO$	Compounds Containing Nitrogen (Amines/Amides)
126	1-Methylsulfinylbutenyl isothiocyante	$C_6H_9NOS_2$	Compounds Containing Nitrogen (Amines/Amides)
127	1-Isothiocyanato-9-(methylsulfinyl)-nonane	$C_{11}H_{21}NOS_2$	Compounds Containing Nitrogen (Amines/Amides)
128	8-acetyl-5,7-dimethoxy-2,2-dimethylchromene	$C_{15}H_{18}O_4$	Phenolic
129	Coralyne	$C_{21}H_{22}O_5$	Flavonoid
130	legonoxamine E	$C_{26}H_{40}N_4O_8$	Amino Acids / Peptides
131	PHENYLETHANOLAMINE	$C_8H_{11}NO$	Compounds Containing Nitrogen (Amines/Amides)
132	Methyleugenol	$C_{11}H_{14}O_2$	Phenolic
133	Kirenol	$C_{20}H_{28}O_3$	Diterpenoid

134	Quercetin-7-O-beta-D-glucopyranoside	$C_{21}H_{20}O_{12}$	Flavonoid Glikosida
135	Baccatin III	$C_{31}H_{38}O_{11}$	Triterpenoid
136	1-METHYL-6,7-DIHYDROXY-1,2,3,4-TETRAHYDROISOQUINOLINE	$C_{10}H_{13}NO_2$	Alkaloid / Heterocyclic Base
137	15-Acetoxyeremantholide B	$C_{22}H_{28}O_8$	Other Secondary Metabolites
138	bonactin	$C_{23}H_{28}O_7$	Flavonoid Glikosida
139	isoabienol	$C_{20}H_{34}O$	Diterpenoid
140	Stigmasterol	$C_{16}H_{22}O_{10}$	Asam Organik Alifatik
141	acanthoside B	$C_{28}H_{36}O_{13}$	Flavonoid Glikosida
142	Peonidin-3-O-glucoside	$C_{22}H_{23}O_{11}$	Flavonoid Glikosida
143	Ferulic acid	$C_{10}H_{10}O_4$	Asam Fenolat
144	CAFFEATE	$C_9H_8O_4$	Asam Fenolat
145	Eupalinilide C	$C_{20}H_{31}NO_7$	Alkaloid / Heterocyclic Base
146	9-Methoxy-2,2-dimethyl-2,6-dihydro-pyrano[3,2-c]quinolin-5-one	$C_{15}H_{15}NO_3$	Alkaloid / Heterocyclic Base
147	2-Deacetoxy taxinine B	$C_{35}H_{42}O_9$	Triterpenoid
148	3-Hydroxyphysodic acid	$C_{26}H_{30}O_9$	Flavonoid
149	SALICYLIC ACID	$C_7H_6O_3$	Asam Fenolat
150	Chafuroside A	$C_{29}H_{48}O$	Other Secondary Metabolites
151	Rhoifolin	$C_{27}H_{30}O_{14}$	Flavonoid Glikosida
152	13OH-terracinolide B	$C_{38}H_{52}O_{18}$	Other Secondary Metabolites
153	Geniposidic acid	$C_{27}H_{41}NO_2$	Alkaloid / Heterocyclic Base
154	Bixin	$C_{21}H_{20}O_{10}$	Flavonoid Glikosida
155	Kuwanon E	$C_{25}H_{26}O_6$	Flavonoid
156	1,7-bis(3,4-dihydroxyphenyl)heptan-3-one	$C_{23}H_{30}N_2O_2$	Alkaloid (Indole)
157	Boldine	$C_{20}H_{23}NO_4$	Alkaloid / Heterocyclic Base
158	brucine	$C_{16}H_{24}O_{11}$	Aliphatic Organic Acids
159	Brusatol	$C_{26}H_{32}O_{11}$	Other Secondary Metabolites

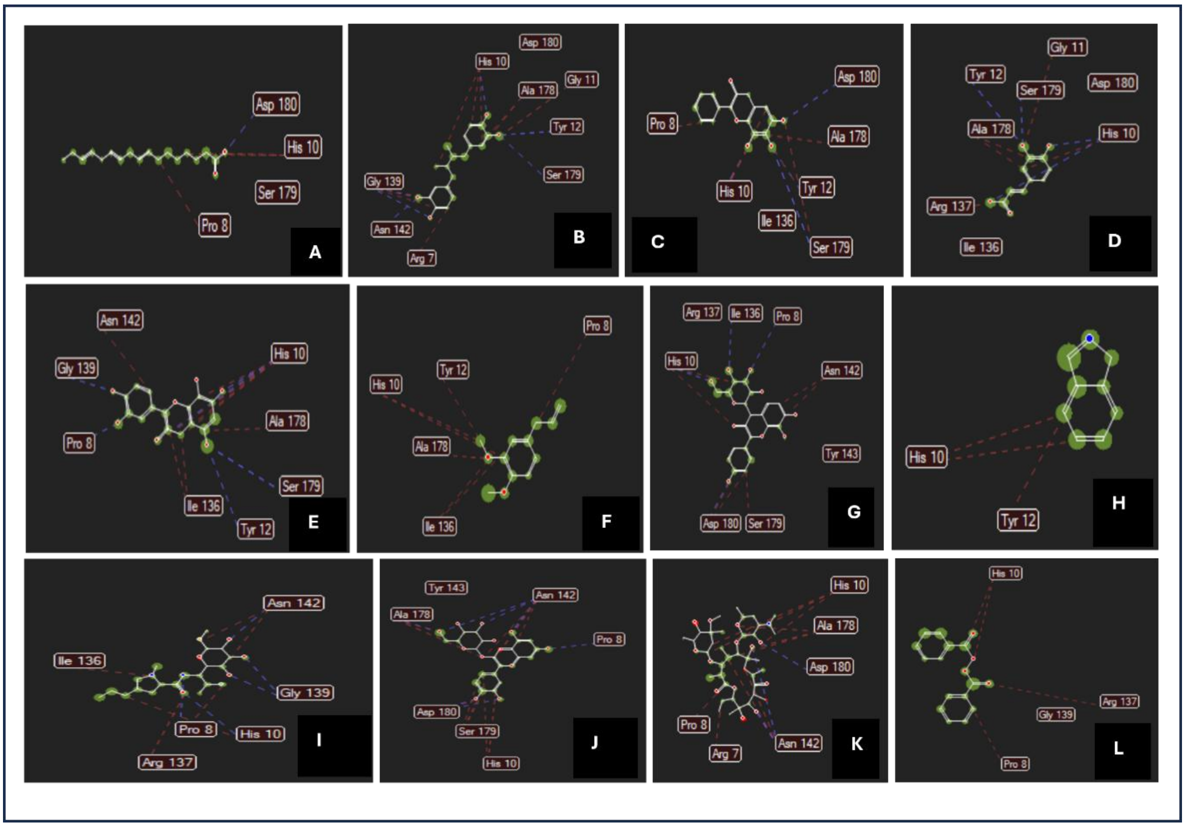

Supplementary Figure S2. Two-dimensional interaction maps of the ligand–receptor complexes against *C. acnes*: (A) Linolenic acid, (B) NDGA, (C) Baicalin, (D) Caffeate, (E) Quercetin, (F) Methyleugenol, (G) Vitexin, (H) Indole, (I) Clindamycin, (J) Cyanidin-3-O-galactoside, (K) Erythromycin, and (L) Benzoyl peroxide. Blue dashed lines indicate hydrogen bonds, whereas red dashed lines represent steric (van der Waals) interactions.

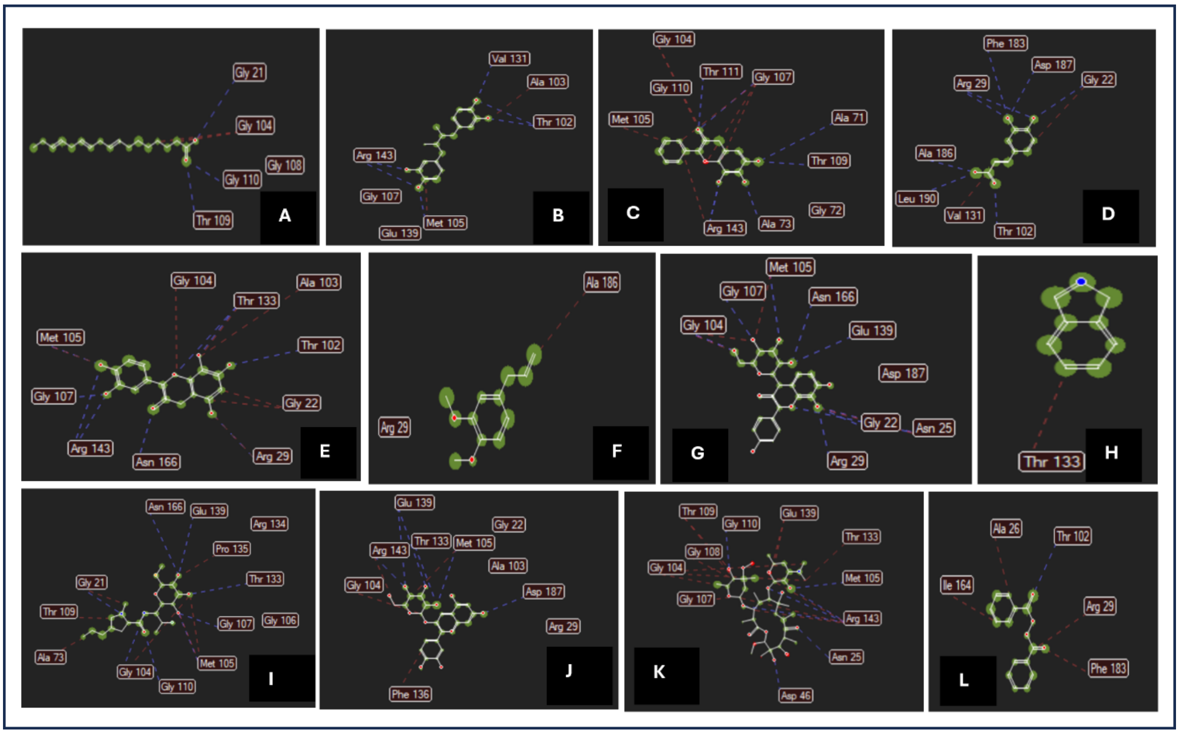

Supplementary Figure S3. Two-dimensional interaction maps of the ligand–receptor complexes against *S. epidermidis*: (A) Linolenic acid, (B) NDGA, (C) Baicalin, (D) Caffeate, (E) Quercetin, (F) Methyleugenol, (G) Vitexin, (H) Indole, (I)

Clindamycin, (J) Cyanidin-3-O-galactoside, (K) Erythromycin, and (L) Benzoyl peroxide. Blue dashed lines indicate hydrogen bonds, whereas red dashed lines represent steric (van der Waals) interactions.

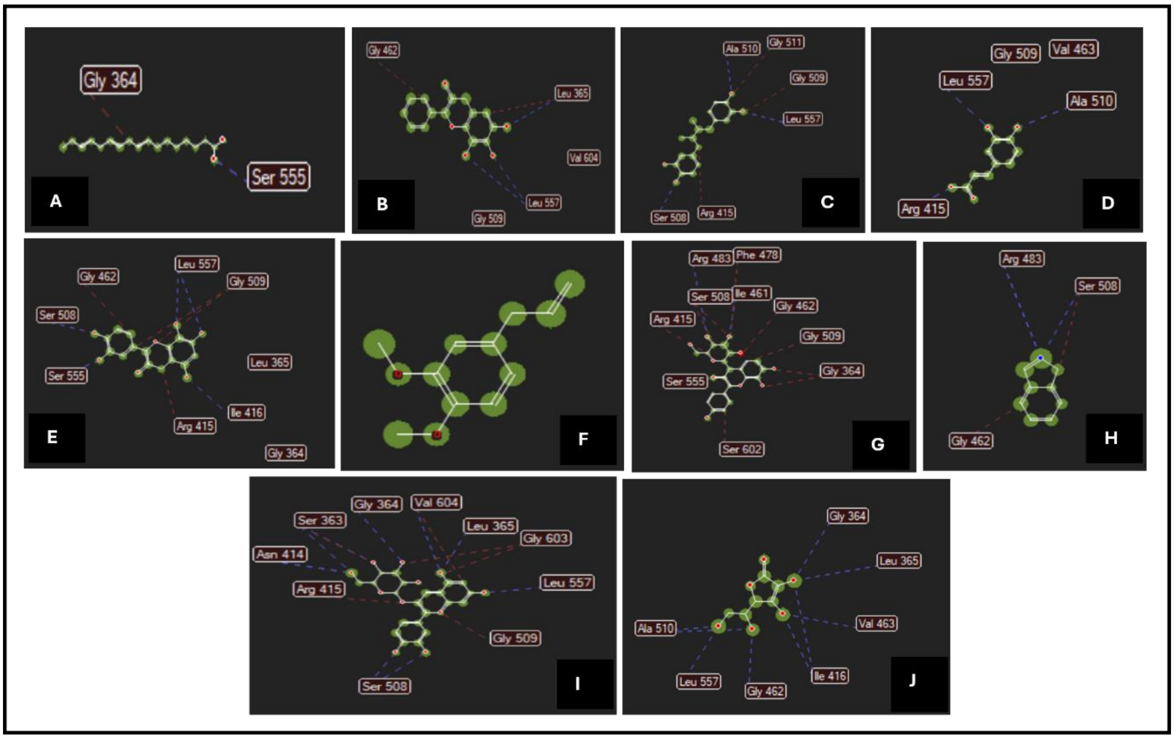

Supplementary Figure S4. Two-dimensional interaction maps of the ligand–receptor complexes against antioxidants: (A) Linolenic acid, (B) Baicalin, (C) NDGA, (D) Caffeate, (E) Quercetin, (F) Methyleugenol, (G) Vitexin, (H) Indole, (I) Cyanidin-3-O-galactoside, and (J) Ascorbic acid. Blue dashed lines indicate hydrogen bonds, whereas red dashed lines represent steric (van der Waals) interactions.

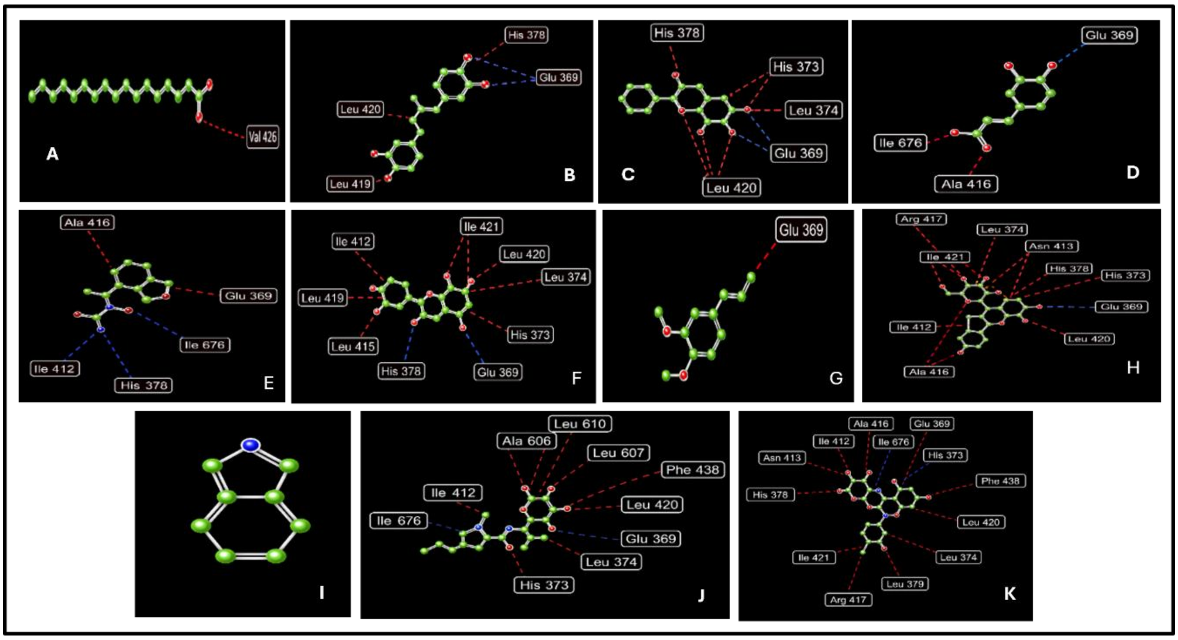

Supplementary Figure S5. Two-dimensional interaction maps of ligand and receptor complexes against 15-LOX: (A) Linolenic acid, (B) NDGA, (C) Baicalin, (D) Caffeate, (E) Zileuton, (F) Quercetin, (G) Methyleugenol, (H) Vitexin, (I) Indole, (J) Clindamycin, (K) Cyanidin-3-O-galactoside. Blue dashed lines indicate hydrogen bonds, whereas red dashed lines represent steric (van der Waals) interactions.
